# VEXAS syndrome is characterized by inflammasome activation and monocyte dysregulation

**DOI:** 10.1038/s41467-024-44811-4

**Published:** 2024-01-30

**Authors:** Olivier Kosmider, Céline Possémé, Marie Templé, Aurélien Corneau, Francesco Carbone, Eugénie Duroyon, Paul Breillat, Twinu-Wilson Chirayath, Bénédicte Oules, Pierre Sohier, Marine Luka, Camille Gobeaux, Estibaliz Lazaro, Roderau Outh, Guillaume Le Guenno, François Lifermann, Marie Berleur, Melchior Le Mene, Chloé Friedrich, Cédric Lenormand, Thierry Weitten, Vivien Guillotin, Barbara Burroni, Jeremy Boussier, Lise Willems, Selim Aractingi, Léa Dionet, Pierre-Louis Tharaux, Béatrice Vergier, Pierre Raynaud, Hang-Korng Ea, Mickael Ménager, Darragh Duffy, Benjamin Terrier

**Affiliations:** 1 Université de Paris Cité, Institut Cochin, CNRS UMR8104, INSERM U1016, Paris, France; 2 Hematology Laboratory, Assistance Publique-Hôpitaux de Paris, Centre-Université de Paris Cité, Cochin Hospital, Paris, France; 3 Institut Pasteur, Université de Paris Cité, Translational Immunology Unit, Paris, France; 4 Sorbonne Université, Faculté de Médecine, UMS037, PASS, Plateforme de Cytométrie de la Pitié-Salpêtrière CyPS, Paris, France; 5 Université de Paris Cité, Imagine Institute, Laboratory of Inflammatory Responses and Transcriptomic Networks in Diseases, Atip-Avenir Team, INSERM UMR 1163, Paris, France; 6 Labtech Single-Cell@Imagine, Imagine Institute, INSERM UMR 1163, Paris, France; 7 Université de Paris Cité, INSERM, U970, PARCC, F-, Paris, France; 8 Department of Internal Medicine, National Reference Center for Rare Systemic Autoimmune Diseases, AP-HP, APHP-CUP, Hôpital Cochin, Paris, France; 9 Université de Paris Cité, INSERM, UMR-S 1132 BIOSCAR, Paris, France; 10 Department of Pathology, AP-HP, APHP-CUP, Hôpital Cochin, Paris, France; 11 Biochemistry Laboratory, Assistance Publique-Hôpitaux de Paris, Centre-Université de Paris Cité, Cochin Hospital, Paris, France; 12 Department of Internal Medicine, Bordeaux University Hospital-Haut-Lévêque, Pessac, France; 13 Department of Internal Medicine, Centre Hospitalier de Perpignan, Perpignan, France; 14 Department of Internal Medicine, Clermont-Ferrand University Hospital, Clermont-Ferrand, France; 15 Department of Internal Medicine, Côte-d’Argent Hospital, Dax, France; 16 Department of Internal Medicine, AP-HP, APHP-NUP, Hôpital Bichat, Paris, France; 17 Université de Strasbourg, Department of Dermatology, CHRU Strasbourg, Strasbourg, France; 18 Department of Internal Medicine, Centre Hospitalier (CHICAS), Gap, France; 19 Department of Internal Medicine, Bordeaux University Hospital-Saint-André, Bordeaux, France; 20 Sorbonne University – 47-83 Boulevard de l’Hopital, Paris, France; 21 Hematology Department, Assistance Publique-Hôpitaux de Paris, Centre-Université de Paris Cité, Cochin Hospital, Paris, France; 22 Dermatology Department, Assistance Publique-Hôpitaux de Paris, Centre-Université de Paris Cité, Cochin Hospital, Paris, France; 23 Pathology Department, Bordeaux University Hospital-Haut-Lévêque, Pessac, France; 24 Pathology Department, Centre Hospitalier de Perpignan, Perpignan, France; 25 Rheumatology Department, AP- HP, Lariboisière Hospital, Paris, France

**Keywords:** Myelopoiesis, Inflammasome, Immunological disorders, Inflammation

## Abstract

Acquired mutations in the *UBA1* gene were recently identified in patients with severe adult-onset auto-inflammatory syndrome called VEXAS (vacuoles, E1 enzyme, X-linked, autoinflammatory, somatic). However, the precise physiological and clinical impact of these mutations remains poorly defined. Here we study a unique prospective cohort of VEXAS patients. We show that monocytes from VEXAS are quantitatively and qualitatively impaired and display features of exhaustion with aberrant expression of chemokine receptors. In peripheral blood from VEXAS patients, we identify an increase in circulating levels of many proinflammatory cytokines, including IL-1β and IL-18 which reflect inflammasome activation and markers of myeloid cells dysregulation. Gene expression analysis of whole blood confirms these findings and also reveals a significant enrichment of TNF-α and NFκB signaling pathways that can mediate cell death and inflammation. This study suggests that the control of the nflammasome activation and inflammatory cell death could be therapeutic targets in VEXAS syndrome.

## Abstract (web summary)

Acquired mutations of the gene UBA1 occurring in myeloid cells that result in the expression of impaired isoforms of the enzyme E1 have been described in patients with a severe adult onset auto-inflammatory syndrome called VEXAS. Here the authors profile patients with UBA1 mutations presenting with or without VEXAS disease and show VEXAS disease is characterized by inflammasome activation and monocyte dysregulation.

## Introduction

Somatic missense mutations affecting methionine-41 (p.Met41) or splice sites in the *UBA1* gene, which result in the expression of a catalytically impaired isoform of the protein, were recently identified in patients with severe adult-onset autoinflammatory disease^1,2^. The E1 ubiquitin-activating enzyme UBA1 is responsible for initiation of the activation, conjugation and ligation of ubiquitin to target protein substrates, playing a critical role in regulating protein homeostasis and cellular processes^3^. Using a genotype-driven approach from the analysis of peripheral blood exome sequence data, Beck et al. identified a disorder named VEXAS (vacuoles, E1 enzyme, X-linked, autoinflammatory, somatic) syndrome^1,2^.

Prevalence of VEXAS syndrome is not yet known but the disease frequency is likely underestimated. VEXAS syndrome was initially exclusively described in males showing somatic mutations in *UBA1*^1^. However, we and others described VEXAS syndrome in female patients, consequent to acquired somatic mutation and X monosomy in hematological cells^4^. Clinical manifestations include fever, neutrophilic cutaneous lesions, arthralgias, pulmonary inflammation, chondritis, and vasculitis, supporting the recruitment and accumulation of inflammatory cells in these tissues^1,5,6^. Additionally, patients with VEXAS suffer from a spectrum of hematologic manifestations mainly including macrocytic anemia, thrombocytopenia, thromboembolic disease, and progressive bone marrow failure, sharing some features with myelodysplastic syndrome (MDS)^6^. Vacuoles are localized predominantly in promyelocytes, myelocytes and erythroid precursors in the bone marrow from VEXAS patients, but what these vacuoles contain is not yet clear^6^. VEXAS syndrome is commonly refractory to conventional disease-modifying antirheumatic drugs and biological targeted therapies may show partial efficacy, suggesting a highly dysregulated inflammatory response. The hypomethylating agent azacytidine, that can be effective in MDS associated or not with inflammatory diseases, showed some efficacy in VEXAS patients^7^, as well as ruxolitinib, a potent and selective oral inhibitor of both JAK1 and JAK2 protein kinases^8^. So far, the only curative option in VEXAS syndrome with severe manifestations was reported to be allogeneic stem cell transplantation^9^.

Somatic mutations affecting the p.Met41 were described in hematopoietic stem cells and peripheral blood myeloid cells but not in mature lymphocytes nor fibroblasts. Mutant cells showed decreased ubiquitylation activating cellular stress responses that lead to upregulation of the unfolded-protein response (UPR), dysregulation of autophagy, and a shared gene expression signature consistent with the activation of multiple innate immune pathways^1^. However, little is known about more detailed immunological features and the molecular mechanisms associated with this impaired ubiquitylation.

Our hypothesis-driven approach was that the mutation of the *UBA1* gene, by causing a disruption of the UPR response and dysregulation of autophagy, as suggested in the first study describing VEXAS syndrome, would induce an excessive inflammatory response through an increase in programmed inflammatory cell death.

To explore the inflammatory mechanisms associated with *UBA1* somatic mutations and to identify therapeutic targets, we used an integrative approach based on clinical and biological data, in-depth phenotypical analysis of whole blood immune cells, cytokine profiling, whole blood bulk RNA analysis, single-cell RNA sequencing of peripheral blood mononuclear cells (PBMCs), and tissue imaging by mass cytometry on skin biopsies, on a large group of *UBA1*-mutated individuals with autoinflammatory disease (VEXAS) in comparison to autoinflammatory individuals without *UBA1* mutation (VEXAS-like), low-risk MDS without features of inflammation and aged gender-matched healthy individuals. We demonstrate that circulating monocytes from *UBA1*-mutated individuals, when compared with VEXAS-like, MDS and healthy controls, were quantitatively and qualitatively impaired and displayed features of exhaustion associated with aberrant expression of chemokine receptors, and dysregulation of IL-1β and IL-18 processing consistent with activation of the inflammasome pathway. Transcriptomic analyses also revealed a significant enrichment of TNF-α and NFκB signaling that could mediate cell death and inflammation. Within affected tissues bearing *UBA1* somatic mutations, CD16^+^ CD163^+^ monocytes and M1 macrophages were abundantly present, and gene expression analysis on skin biopsy confirmed the upregulation of the *IL1B* gene and IL-1β pathway. We identified at the single cell level molecular pathways involved in monocyte dysregulation, especially a defective TYROBP/DAP12 and β-catenin signaling pathway and the activation of proinflammatory programmed cell death pathways.

## Results

### Characteristics of the cohort

**Fig. 1:**
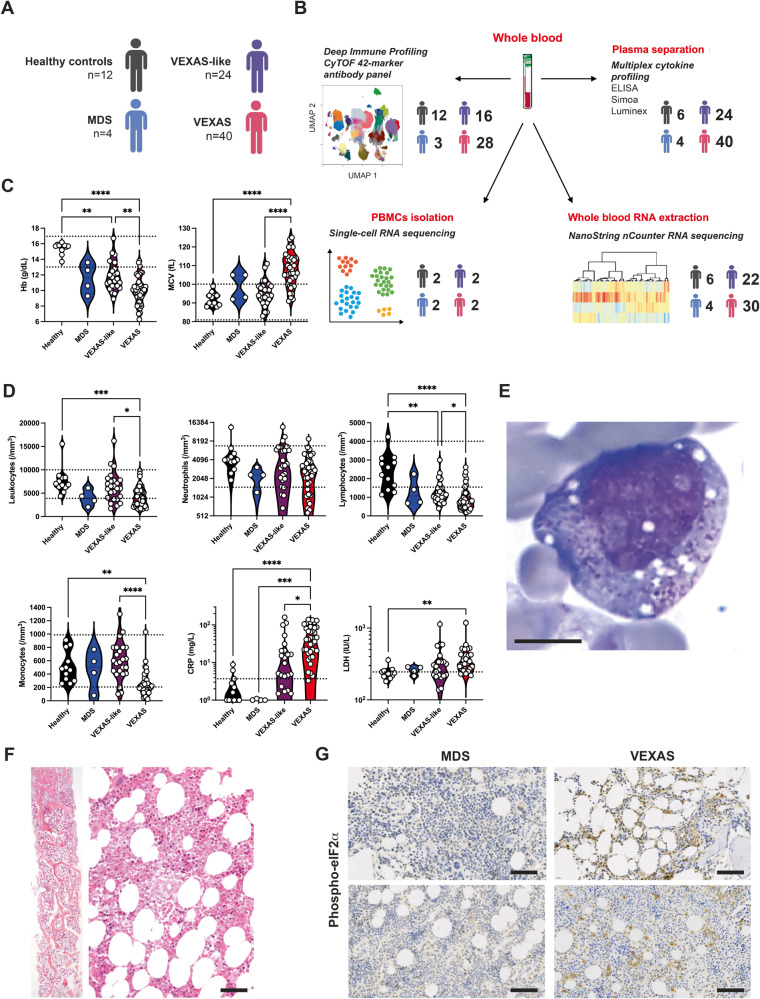
Clinical and laboratory findings in patients with VEXAS syndrome, VEXAS-like, MDS and healthy controls. **A**, **B** 40 individuals with VEXAS syndrome, 26 individuals with VEXAS-like, 4 with MDS and 12 aged gender-matched healthy controls were included, and samples were assessed using deep immune profiling, plasma multiplex cytokine profiling, whole blood RNA extraction and single-cell RNA sequencing. **C** Hemoglobin (Hb) and mean corpuscular volume (MCV) from patients with VEXAS syndrome, VEXAS-like, MDS and healthy controls (Hb, ****P  <  0.0001, **P  =  0.0068 and **P = 0.0048; MCV, ****P < 0.0001). Each dot represents a single patient. **D** Leukocytes, neutrophils, lymphocytes and monocytes count, C-reactive protein (CRP) and lactate dehydrogenase (LDH) from patients with VEXAS, VEXAS-like, MDS and healthy controls (leukocytes, ***P  =  0.0008 and *P  =  0.0202; lymphocytes, ****P  <  0.0001, **P  =  0.0049 and *P  =  0.0163; monocytes, ****P  <  0.0001 and **P  =  0.0039; CRP, ****P  <  0.0001, ***P  =  0.0006 and *P = 0.0139; LDH, **P = 0.0017). Each dot represents a single patient. **E** Bone marrow aspiration showing vacuoles restricted to myeloid and erythroid precursor cells in a VEXAS patient (MGG staining, ×100, scale bar 10 μm). **F** Bone marrow biopsy from a VEXAS patient showing overrepresentation of the myeloid cell lineage with mature and immature forms (Magnification ×20, scale bar 100 μm). **G** Immunohistochemical staining of phosphorylated eIF2α on bone marrow biopsy from MDS and VEXAS patients, showing expression of p‑EIF2α only in VEXAS (Magnification ×20, scale bar 100 μm). *P* values were determined by the two-sided Kruskal-Wallis test, followed by Dunn’s post test for multiple group comparisons. *P < 0.05; **P < 0.01; ***P < 0.001, ****P < 0.0001.

We analyzed the immune response elicited by *UBA1* somatic mutations in a unique cohort of individuals with severe autoinflammatory diseases showing *UBA1* mutation (VEXAS) (*n* = 40) including 2 patients with low variant allele frequency (VAF) of *UBA1* mutation, non-mutated aged gender-matched individuals with severe autoinflammatory diseases (VEXAS-like) (*n* = 24), low-risk MDS (*n* = 4) and aged gender-matched used as healthy controls (n = 12) (Fig. 1A, Supplementary Table 1 and Supplementary Fig. 1). In all cases, fresh material was used to analyze *UBA1* mutations and to generate multi-Omics data including characterization of associated myeloid mutations by Next-Generation Sequencing (NGS) technology (Fig. 1B and Supplementary Fig. 1). We found at least one additional mutation in 18 VEXAS patients, including *DNMT3A* mutation in 11 cases. No other mutations other than those affecting p.Met41 or the splice site were detected in our cohort. The two patients with a low VAF of *UBA1* mutation (9% and 6%) did not differ from patients with higher VAF in terms of clinical manifestations. Similar results were very recently described in a large cohort of 77 VEXAS patients^10^.

All individuals were male with a median age of 74 (55–90) years for VEXAS, 70 (54–82) years for VEXAS-like, 76 (70–88) years for MDS and 60 (55–85) years for healthy controls at time of sampling. Each of the VEXAS individuals had either one of the three somatic variants of codon 41 in *UBA1* (p.Met41Thr in 18, p.Met41Val in 10, or p.Met41Leu in 6) or a splice mutation affecting the p.Met41 in 6 cases. The majority of VEXAS individuals had recurrent fevers, arthralgias/arthritis, neutrophilic dermatosis and cutaneous vasculitis, pulmonary involvement (Supplementary Table 1), macrocytic anemia (Fig. 1C), leukopenia, mainly lymphocytopenia and monocytopenia, and increased C-reactive protein and lactate dehydrogenase levels (Fig. 1D), and bone marrow vacuolization restricted to myeloid and erythroid precursor cells (Fig. 1E). VEXAS-like patients have previously been diagnosed as relapsing polychondritis, Sweet’s syndrome, polyarteritis nodosa and giant cell arteritis/polymyalgia rheumatica, but all had either atypical manifestations for these diagnoses and/or highly refractory disease. Bone marrow biopsies from VEXAS patients (*n* = 5), compared with VEXAS-like (*n* = 2) and MDS (*n* = 3), showed heterogeneity in morphology and cellularity but predominantly over-representation of the myeloid cell lineage with many mature and immature forms, and slight abnormalities of erythroblasts and megakaryocytes (example from a VEXAS patient shown in Fig. 1F). Myeloid cells in bone marrow from VEXAS patients only, but not in VEXAS-like nor MDS patients, showed expression of phosphorylated eIF2α (p‑EIF2α) confirming the activation of cellular stress responses leading to upregulation of the UPR and dysregulation of autophagy as described before^1^ (Fig. 1G).

### An aberrant phenotype of circulating monocytes in *UBA1*-mutated individuals

**Fig. 2:**
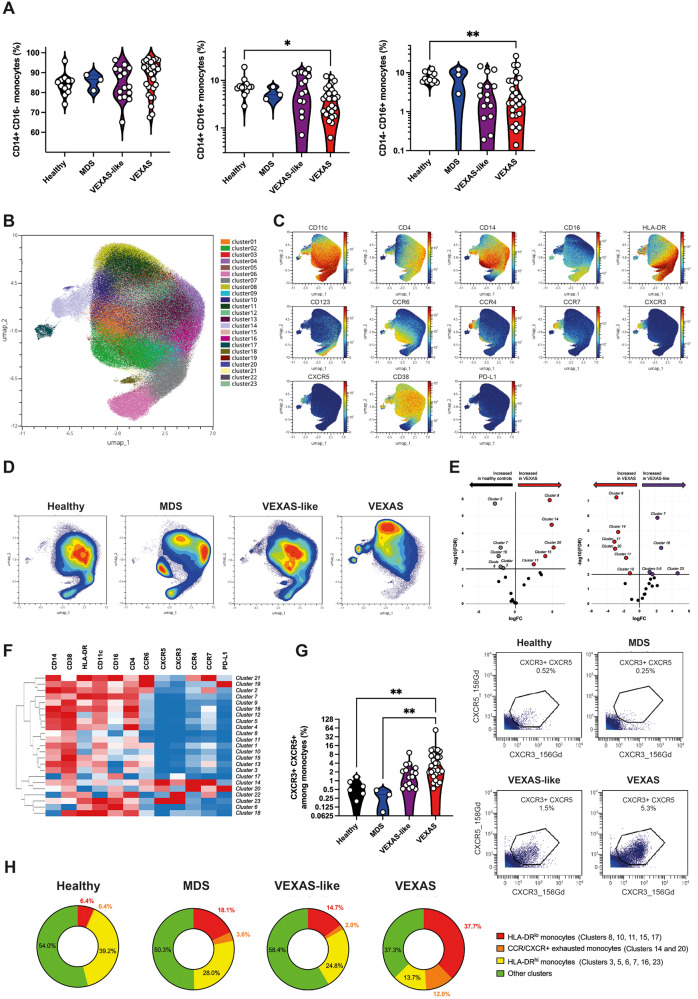
Phenotyping of peripheral blood monocytes in VEXAS syndrome. **A** Proportions (frequencies) of classical (CD14^+^, CD16^−^), intermediate (CD14^+^, CD16^+^), and nonclassical (CD14^lo^, CD16^+^) monocytes among blood monocytes from VEXAS patients, VEXAS-like patients, MDS and healthy controls, were analyzed (CD14^+^, CD16^+^, *P  =  0.00379; CD14^lo^, CD16^+^, **P  =  0.0084). Each dot represents a single patient. **B** Non-supervised Uniform Manifold Approximation and Projection (UMAP) of blood monocytes. Cells are automatically separated into spatially distinct subsets according to the combination of markers that they express. **C** UMAP of blood monocytes stained with 13 markers and measured with mass cytometry. **D** UMAP colored according to cell density across patients’ groups. Red indicates the highest density of cells. **E** Volcan plots of differentially represented monocyte subsets showing clusters increased or decreased in VEXAS patients compared to healthy controls, and VEXAS patients compared to VEXAS-like. **F** Heatmap representation of all monocyte clusters, ordered by hierarchical clustering and expression of the cell surface markers. **G** Proportion (frequencies) of CXCR3 + CXCR5+ expressing monocytes. Each dot represents a single patient. Gating strategy for the analysis of expression of CXCR3 and CXCR5 on blood monocytes, Illustrative dot plots are shown and enumeration of percentages of CXCR3 + CXCR5+ cells (**P  =  0.0012 and **P  =  0.0048). **H** Pie chart showing the proportion of HLA-DR^lo^ dysfunctional monocytes, exhausted monocytes expressing chemokine receptors, HLA-DR^hi^ functional monocytes, and other clusters not significantly different between groups. Numbers on the pie charts indicate the median proportion of each monocytes subsets in each group. *P* values were determined by the two-sided Kruskal-Wallis test, followed by Dunn’s post test for multiple group comparisons. *P < 0.05; **P < 0.01; ***P < 0.001, ****P < 0.0001.

We first performed a multiparameter phenotyping of peripheral blood leukocytes in individuals using mass cytometry (Supplementary Table 2). Gating strategy for monocytes is described in Supplementary Fig. 2A and all the individual data are available in Supplementary Data 1. Immunoprofiling of peripheral blood from VEXAS patients revealed a significant decrease in circulating monocytes, primarily involving intermediate (CD14^+^ CD16^+^) and nonclassical (CD14^lo^ CD16^+^) monocytes (Fig. 2A).

By integrating expression data of surface markers (i.e. HLA-DR, CD11c, CD14, CD16, CD4, CD38, CD123, CCR4, CCR6, CCR7, CXCR3, CXCR5, PD-L1) from patients with VEXAS, VEXAS-like, MDS and healthy controls and subjecting them to dimensionality reduction using non-supervised Uniform Manifold Approximation and Projection (UMAP)^11^ (Fig. 2B, C), we analyzed monocyte subsets from all patient groups. Analysis revealed significant differences in the repartition of monocyte subsets in VEXAS in comparison with other groups (Fig. 2D and Supplementary Fig. 2B). Volcano plots of differentially represented monocyte clusters showed an increased proportion of clusters 8, 11, 14, 15 and 20 in VEXAS compared to healthy controls, and a decreased proportion of clusters 5, 6, 7, 13 and 16 (Fig. 2E and Supplementary Fig. 2B). Heatmap representing the relative expression of cell surface markers identified clusters 8, 11, 14, and 15 as dysfunctional HLA-DR^lo^ classical monocytes, and cluster 20 as exhausted HLA-DR^lo^ CD38^+^ PD-L1^hi^ nonclassical monocytes (Fig. 2F and Supplementary Fig. 3). Cell surface expression of HLA-DR on monocytes, evaluated by mean metal intensity (MMI), was also significantly decreased in VEXAS patients (Supplementary Fig. 4B). Clusters 5, 6, 7, 13, and 16, significantly decreased in VEXAS, identified in contrast functional HLA-DR^hi^ classical, intermediate and nonclassical monocytes (Fig. 2F). Similarly, compared to VEXAS-like, VEXAS patients had an increased proportion of the same dysfunctional classical and nonclassical monocytes and exhausted HLA-DR^lo^ CD38^+^ PD-L1^hi^ nonclassical monocytes, while the same functional HLA-DR^hi^ monocytes were decreased (Fig. 2E, F, Supplementary Fig 4). Consistent with these findings, whole blood expression of activation-related genes^12^—such as *HLADRB1* and *CD86*—were significantly decreased, and expression of exhaustion-related genes^13^—such as *PLAUR and RELB*—were significantly increased in VEXAS patients compared to other groups (Supplementary Fig. 4C).

VEXAS syndrome has been associated with various clinical manifestations, related to massive influx of innate immune cells. We analyzed the expression of chemokines receptors on monocytes. Dysfunctional and exhausted monocytes from VEXAS (clusters 14 and 20) showed significantly higher expression of CXCR3, CXCR5, CCR4 and CCR7 compared to VEXAS-like, MDS and healthy controls (Fig. 2G and Supplementary Fig. 4D). These cells expressed low levels of CD14 supporting that they were nonclassical monocytes. Interestingly, CXCR3 expression is able to promote transendothelial migration of cells to sites of inflammation, through the interaction of CXCR3 with CXCL9, 10, and 11. CXCR3 and CCR4 were also shown to be expressed by immune cells in inflamed skin tissue, both receptors having been associated with dermal recruitment of immune cells, an important finding given the high frequency of skin lesions in VEXAS^14^. Also, CXCR5, a receptor for CXCL13, and CCR7, a receptor for CCL19 and CCL21, are key regulators of cell localization in secondary lymphoid organs^15^, which could explain monocyte migration to lymph nodes and spleen. Monocyte chemotactic factor chemokine (C-C motif) ligand 2 (CCL2), also called MCP-1, was increased only in the blood of VEXAS patients, whereas transcripts of its receptor *CCR2* were significantly decreased (Supplementary Fig. 4E, F). Such discrepancy between CCR2 expression and CCL2/MCP-1 protein levels was previously shown to represent a feedback mechanism in the regulation of the chemotactic response of monocytes/macrophages^16^.

Altogether, these data suggest that changes in the abundance and phenotype of monocyte subsets within the peripheral blood cell population, with significantly reduced monocytes enriched in dysfunctional and exhausted subsets showing elevated chemokine receptor expression (Fig. 2H and Supplementary Fig. 5A, B), characterize VEXAS patients. Significant decreases in circulating monocytes could suggest either an increased cell death and/or an enhanced monocyte migration into inflamed tissues.

**Fig. 3:**
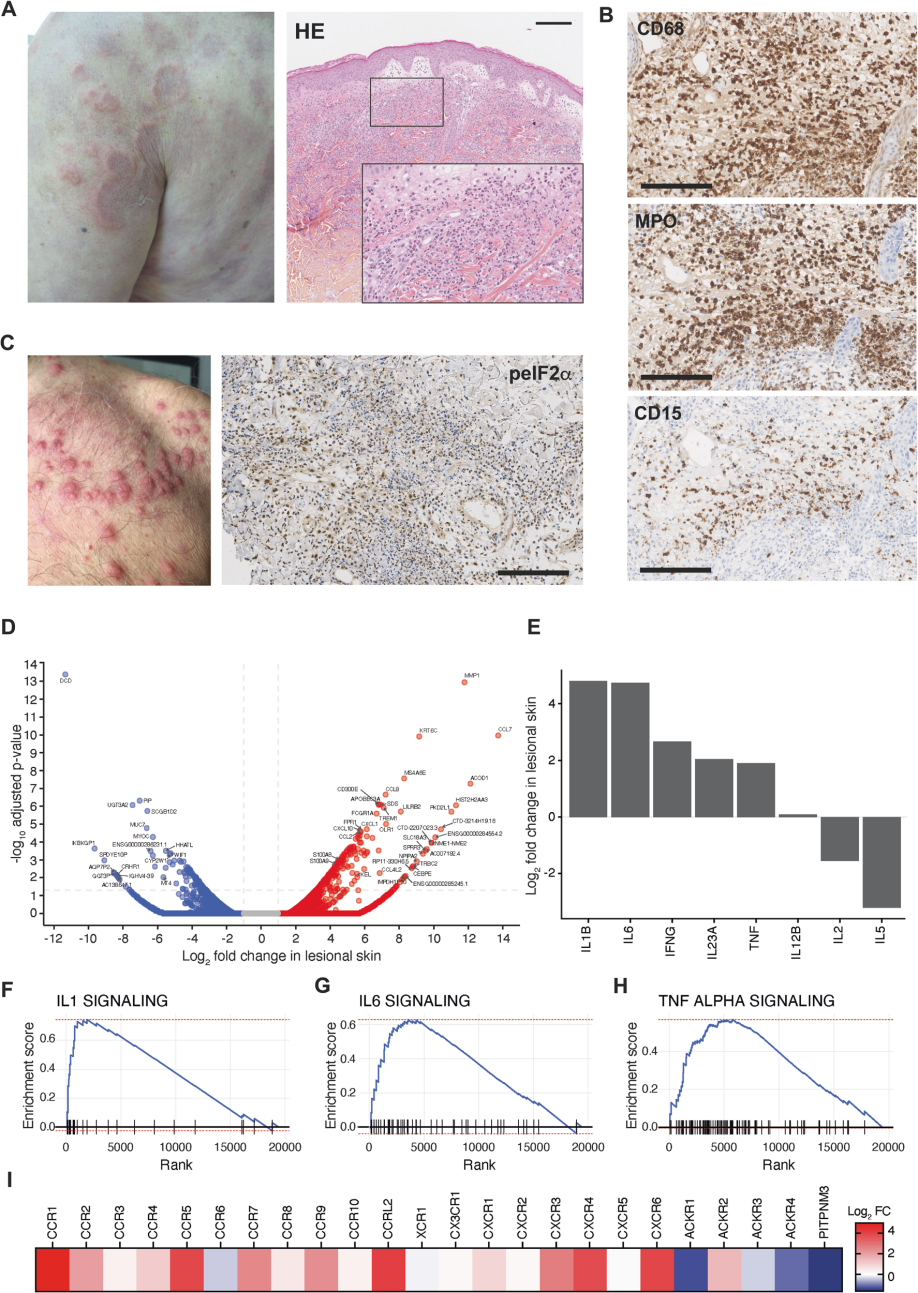
Characterization and anatomical localization of myeloid cells within skin lesion from VEXAS patients. **A** Picture of neutrophilic dermatosis and hematoxylin-eosin staining of skin biopsy of a VEXAS patient. Illustrative picture is shown (Magnification × 10 and × 20, scale bar 100 μm). **B** Immunohistochemical staining of CD68, myeloperoxidase (MPO) and CD15 of the same skin biopsy. Illustrative picture is shown (Magnification × 40, scale bar 50 μm). **C** Picture of neutrophilic dermatosis of another VEXAS patient and immunohistochemical staining of phosphorylated eIF2α on skin biopsy from a VEXAS patient, showing expression of p‑EIF2α in skin lesions. Illustrative picture is shown (Magnification ×40, scale bar 50 μm). **D** Ratio average (RA) plot showing the log ratio in lesional skin versus the average log abundance for each gene. **E** Expression of IFN-γ, IL-12B, IL-1β, IL-2, IL-23A, IL-5, IL-6 and TNF in TPM in lesional and non lesional skin from a VEXAS patient. **F**, **G**, **H** Gene set enrichment analysis of IL-1, IL-6 and TNF-a pathways enriched in VEXAS lesional skin versus non lesional skin. **I** Expression of chemokine receptors in VEXAS lesional skin.

We next analyzed skin biopsies from VEXAS patients. Formalin-fixed paraffin-embedded (FFPE) slides from skin biopsy specimens from 3 VEXAS at time of active lesions were analyzed (Fig. 3A). As previously reported^17^, we detected by Sanger sequencing on both paired bone marrow or peripheral blood and skin biopsy specimens from VEXAS patients the same *UBA1* mutation. Immunohistochemical staining showed abundant monocytes expressing CD68 and MPO within the dermis of inflamed skin lesions, whereas neutrophils expressing CD15 were less numerous (Fig. 3B), supporting the potential migration of monocytes into the tissues. As in bone marrow samples, mononuclear cells in the skin showed increased expression of p‑EIF2α consistent with the upregulation of the UPR (Fig. 3C). We then used imaging mass cytometry to determine anatomical localization of inflammatory cells within target tissues from VEXAS patients, using a combination of markers (Supplementary Fig. 6A, B). Focusing on monocytes/macrophages, nonclassical (CD14^low^ CD16^+^) and intermediate (CD14^+^ CD16^+^) monocytes expressing CD163 (Supplementary Fig. 6A, B) were abundant in skin lesions from VEXAS cases, adjacent to blood vessels identified by αSMA and CD31 (Supplementary Fig. 6B, C), suggesting trafficking from the periphery to areas of inflammation. Such a CD16^+^ CD163^+^ monocyte population was described in necrotizing enterocolitis in premature infants as playing an important role in the inflammatory process and trafficking to sites of inflammation, suggesting that these cells could be one of the pathogenic drivers of inflammation in target tissues^18^. Next to CD16^+^ CD163^+^ monocyte-enriched areas, we identified CD68^+^ CD163^-^ macrophages forming clusters or aggregates further away from blood vessels (Supplementary Fig. 6B, D, E). The scavenger receptor CD163 is a highly specific marker for the M2 subpopulation. Accumulation of CD68^+^ CD163^-^ macrophages within skin biopsies suggests abundant proinflammatory M1 macrophages next to CD16^+^ CD163^+^ monocytes. CD68^+^ CD163^-^ macrophages from skin lesions also expressed granzyme B (Supplementary Fig. 6B, F), suggesting that granzyme B may play a role in macrophage functions and pathogenicity associated with skin lesions.

We then performed RNAseq analyses on a lesional skin sample from a single VEXAS patient as part of a personalized medicine protocol.and compared it with the non-lesional skin. Several chemokines, including *CCL2* and *CXCL10*, and *S100A8/S100A9* encoding calprotectin were upregulated in lesional skin (Fig. 3D). We also observed an increase in *IL1B* and *IL6* gene expression in VEXAS skin lesions compared to non-lesional skin (Fig. 3E) and an enrichment in IL-1, IL-6 and TNF-α transcriptional signatures (Fig. 3F, G, H). Finally, we observed an upregulation of *CXCR3* and *CCR7*, suggesting migration of dysfunctional and exhausted monocytes from VEXAS to the skin lesions (Fig. 3I).

Overall, these results obtained without replicates support a scenario in which *UBA1*-mutated inflammatory monocytes aberrantly expressing chemokine receptors could be attracted into target tissues and promote local inflammation.

### VEXAS patients display an increase in proinflammatory cytokines consistent with inflammasome activation

**Fig. 4:**
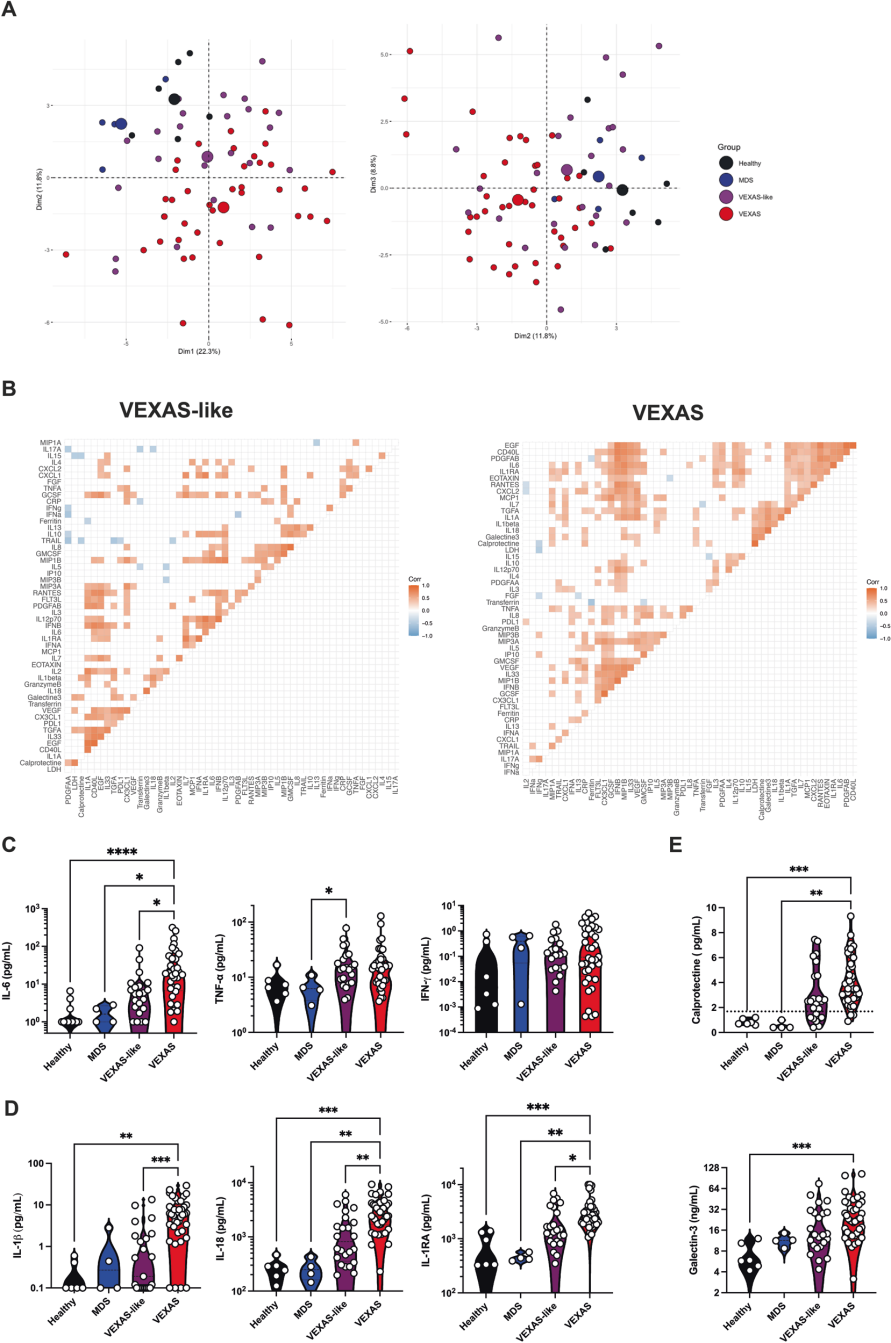
Inflammatory cytokine and chemokines profiling of VEXAS syndrome. **A** Principal component analysis of the cytokine and chemokine data according to dimensions 1, 2 and 3. Median values for each patients’ group are plotted with the large colored circle. Each dot represents a single patient. **B** Correlation matrices across all values of cytokines and chemokines from patient blood, comparing patients with VEXAS and VEXAS-like. Only significant correlations (<0.05) are represented as squares. Pearson’s correlation coefficients from comparisons of cytokine and chemokine measurements within the same patients are visualized by color intensity. **C** IL-6, TNF-α and IFN-γ from patients with VEXAS, VEXAS-like, MDS and healthy controls (IL-6, ****P  <  0.0001, *P  =  0.0156 and *P  =  0.0267; TNF-α, *P  =  0.0377). Each dot represents a single patient. **D** IL-1β, IL-18 and IL-1RA from patients with VEXAS, VEXAS-like, MDS and healthy controls (IL-1β, ***P  =  0.0002 and **P  =  0.0016; IL-18, ***P  =  0.0002, **P  =  0.0022 and **P  =  0.0050; IL-1RA, ***P  =  0.0006, **P  =  0.0016 and *P  =  0.0164). Each dot represents a single patient. **E** Calprotectin and galectin-3 from patients with VEXAS, VEXAS-like, MDS and healthy controls (calprotectin, ***P  =  0.0006 and **P  =  0.0012; galectin-3, ***P  =  0.0009). Each dot represents a single patient. *P* values were determined by the two-sided Kruskal-Wallis test, followed by Dunn’s post test for multiple group comparisons. *P < 0.05; **P < 0.01; ***P < 0.001, ****P < 0.0001.

To further characterize inflammatory responses implicated in VEXAS, we analyzed plasma samples from patients with VEXAS, VEXAS-like, low-risk MDS and healthy controls by Luminex Multiplex assays, conventional and digital ELISA to quantify a total of 52 soluble mediators. Unsupervised principal components analysis (PCA) separated patients with VEXAS syndrome from the other groups on dimension 2, driven by inflammatory cytokines (IL-6, IL-18), IL-1 receptor antagonist (IL-1RA) and myelomonocytic markers (calprotectin and galectin-3) (Fig. 4A).

We next tried to correlate the measurements of these soluble proteins (Fig. 4B). We observed a ‘discriminating VEXAS signature’ that was defined by the following inflammatory mediators, which correlated positively with each other: IL-1α, IL-1β, IL-18, TGF-α, IL-7, galectin-3 and calprotectin. We also observed in VEXAS two additional clusters: the first defined by inflammatory cytokines—IL-6, IL-1RA—and proinflammatory chemokines—CCL2/MCP-1, CCL11/eotaxin, CCL4/MIP-1β, CXCL2, RANTES, PDGF-AB and EGF—and the second defined mainly by proinflammatory chemokines and growth factors—CCL20/MIP-3α, CCL19/MIP-3β, CX3CL1, IL-10, G-CSF, GM-CSF, and VEGF. VEXAS-like patients mainly showed a more heterogeneous signature defined by IL-2, IL-33, and TGF-α, chemokines and growth factors.

As suggested by cytokine measurements and correlation matrix, prominent inflammatory cytokines included IL-6, a key player of exacerbated inflammatory responses, that was significantly increased in VEXAS compared to other groups, whereas tumor necrosis factor–α (TNF-α), another key driver of inflammation, and IFN-γ levels were not significantly elevated (Fig. 4C). IL-1β and IL-18, potent proinflammatory cytokines for which maturation and secretion is governed by the inflammasome^19^, were also significantly increased in VEXAS patients (Fig. 4D). These findings corroborated with the detection of high amounts of circulating IL-1RA, indicating an active antagonism of IL-1 in VEXAS patients (Fig. 4D). We next analyzed the ability of monocytes from VEXAS and VEXAS-like patients to be stimulated in vitro by crystals (i.e. MSU (monosodium urate) and monoclinic calcium pyrophosphate dihydrate and m-CPP (monoclinic calcium pyrophosphate dihydrate) and LPS, both potent activators of the inflammasome, in comparison with aged-patients with gout (Supplementary Fig. 7). In contrast to increased levels of IL-1β in plasma, monocytes from VEXAS patients showed an altered release of IL-1β after stimulation with LPS in comparison with VEXAS-like and gout patients, consistent with the increase of dysfunctional and exhausted monocytes in peripheral blood of VEXAS patients.

Calprotectin and galectin-3 were specifically increased in the peripheral blood of VEXAS patients compared to other groups, highly supporting dysregulation of the myelomonocytic compartment (Fig. 4E). S100A8/S100A9 alarmin calprotectin is known to be released under inflammatory conditions by myeloid cells and promotes NFκB activation^20^ and secretion of multiple inflammatory proteins, such as IL-6^21^. Galectin-3 (formerly known as Mac-2), is secreted from monocytes/macrophages^22,23^ and regulates monocyte/macrophage adhesion, chemotaxis, and apoptosis^24^.

These data highlight broad inflammatory changes, especially involving concomitant release of IL-1β and IL-18 governed by the inflammasome and markers of myeloid cells dysregulation in VEXAS patients.

### Proinflammatory transcriptional signatures characterize VEXAS patients

**Fig. 5:**
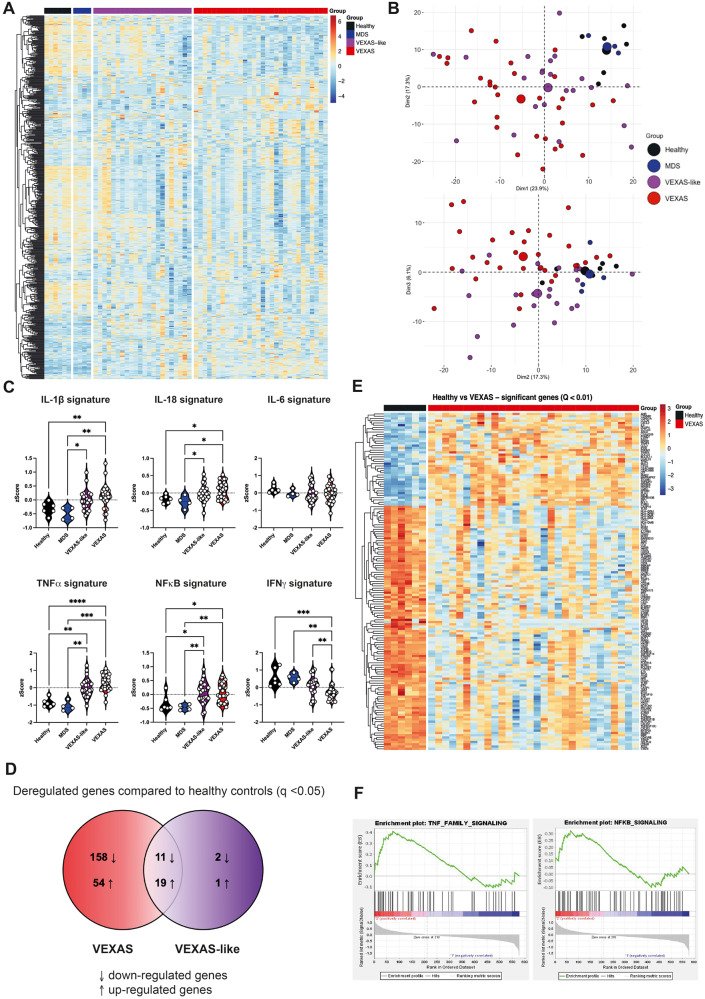
Immunological transcriptional signatures in VEXAS syndrome. **A** Heatmap representation of 579 immunological genes measured by Nanostring approach, ordered by hierarchical clustering in healthy controls (*n* = 6), MDS (*n* = 4), VEXAS-like (*n* = 23), and VEXAS (*n* = 29). **B** Principal component analysis of the transcriptional data according to the 4 groups of patients. Median values for each patients’ group are plotted with the large colored circle. **C** Comparison of IL-1β, IL-18, IL-6, TNF-α, NFκB and type II IFN gene signatures expression in healthy controls, MDS, VEXAS-like and VEXAS patients (IL-1β, **P  =  0.007, **P  =  0.0032 and *P  =  0.0387; IL-18, *P  =  0.0189, *P  =  0.0107 and *P  =  0.0491; TNF-α, ****P  <  0.0001, ***P  =  0.0002, **P  =  0.0071 and **P  =  0.0092; NFκB, **P  =  0.0039, **P  =  0.0058, *P  =  0.0117 and *P  =  0.0180; IFN-γ, ***P  =  0.0010; **P  =  0.0029 and **P  =  0.0096). Each dot represents a single patient. **D** Venn diagram representation showing significant differential gene expression in VEXAS or VEXAS-like patients versus healthy controls. **E** Heatmap representation of differentially expressed genes (q-value < 0.01) in healthy controls (*n* = 6) and VEXAS (*n* = 29) patients, ordered by hierarchical clustering. **F** Gene set enrichment analysis of pathways enriched in VEXAS versus healthy controls, i.e., TNF-α signaling and NFκB signaling. *P* values were determined by the two-sided Kruskal-Wallis test, followed by Dunn’s post test for multiple group comparisons. *P < 0.05; **P < 0.01; ***P < 0.001, ****P < 0.0001.

To investigate the immunological transcriptional signatures that characterize VEXAS patients, we quantified the expression of 594 immune-related genes in peripheral blood cells by using the Nanostring Human Immunology v2 panel kit. Supervised hierarchical clustering showed differences in gene expression between aged gender-matched healthy controls and MDS in one hand, and VEXAS and VEXAS-like cases on the other hand (Fig. 5A). Unsupervised principal components analysis (PCA) also showed distinct clustering of VEXAS syndrome from the other groups (Fig. 5B).

Guided by previous results from plasma cytokine measurement (Fig. 4C, D), we used specific lists of genes to calculate zScores for each transcriptional signature, i.e. type II IFN, TNF-α, IL-1β, IL-18, IL-6, and NFκB (type II IFN, TNF-α, and IL-1β signatures were based on previous data from Urrutia et al.^25^, while IL-18, IL-6 and NFκB signatures were based on the Nanostring Immunology panel annotation file or information from other databases, Supplementary Table 3). IL-1β, IL-18, TNF-α and NFκB signatures were upregulated in both VEXAS and VEXAS-like patients, whereas the IL-6 signature was not significantly different between groups, and the type II IFN (IFN-γ) signature was significantly reduced in VEXAS cases compared to other groups (Fig. 5C).

We then used Venn diagram representation to visualize distinction between gene expression data from VEXAS, VEXAS-like and healthy controls (MDS patients were not included in this analysis due to the small number of patients). We compared the list of significantly dysregulated genes (Mann-Whitney test, q-value < 0.05) in the respective VEXAS and VEXAS-like analyses versus healthy controls (Fig. 5D). Among the 237 genes dysregulated in VEXAS compared to controls, 202 were specifically deregulated in VEXAS of which 152 were down-regulated and 51 up-regulated. The 10 most down-regulated genes were *CCR2, CSF1R, CMKLR1, CIITA, MSR1, CX3CR1, CASP10, CD86, CISH and CXCR2*, while the 10 most up-regulated genes were *PLAU, NFKBIA, LTF, TNFAIP3, CTSG, CEACAM6, CXCL2, CEACAM8, CD83 and CCL20* (Supplementary Table 4). Notably, 34 of the 237 genes differentially dysregulated between VEXAS and controls were also dysregulated in the VEXAS-like versus controls (top genes in Supplementary Table 5 and all genes in Supplementary Data 2), while only 4 genes were specifically dysregulated in VEXAS-like confirming the greater transcriptional heterogeneity of VEXAS-like patients in our cohort.

Heatmap representation of supervised hierarchical clustering showed differences in gene expression between healthy controls and VEXAS patients (Fig. 5E). Gene set enrichment analysis of pathways enriched between healthy controls and VEXAS showed that genes encoding proteins involved in TNF-α pathway signaling and NF∣B signaling were upregulated in VEXAS (Fig. 5F, Supplementary Fig. 8A, B), while those encoding proteins in adaptive immunity, MHC class II antigen presentation and phagocytosis degradation were downregulated (gene set enrichment analysis enrichment score with *q* value < 0.2).

Overall, our data suggest that VEXAS patients exhibit an upregulation of IL-1β and IL-18 signatures at the transcriptional level suggesting inflammasome activation, and TNF-α family and NFκB signaling that could mediate cell death and inflammation.

### Single-cell RNA sequencing of PBMCs highlights dysregulated proinflammatory and cell death signatures in monocytes from VEXAS syndrome

**Fig. 6:**
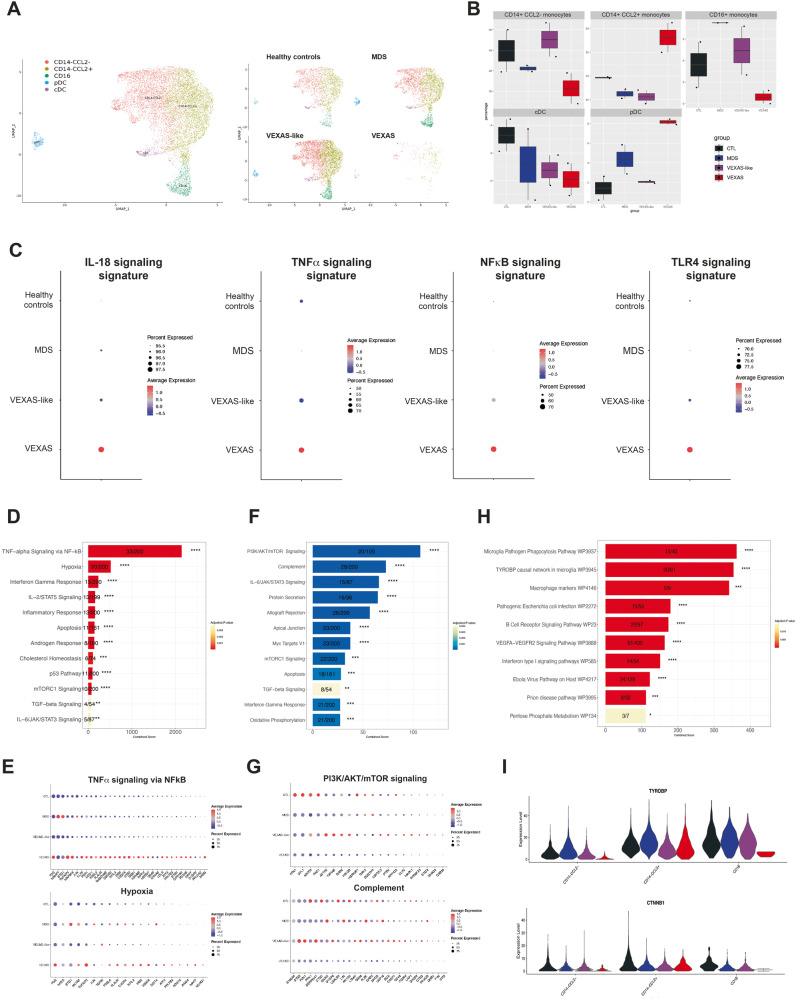
Visual representation of single-cell transcriptomic data of monocytes in VEXAS syndrome. **A** UMAP plots showing the projection of single myeloid cells from PBMCs from patients with VEXAS (*n* = 2), VEXAS-like (*n* = 2), MDS (*n* = 2) and healthy controls (*n* = 2). **B** Proportion (frequencies) of the myeloid cell subsets, i.e. CD14 + CCL2+ monocytes, CD14 + CCL2- monocytes, CD16+ monocytes, cDC and pDC from each patients’ group. Box plots indicate here the minimum value, the first quartile, the median, the third quartile, and the maximum value. **C** IL-18, TNF-α, NFκB and TLR4 signaling Gene expression signatures in monocytes from each patients’ group. The size of the dot represents the percentage of cells in the clusters expressing the gene expression signature and the color intensity represents the average expression of the signature in that cluster. **D** Enriched GO functions of up-regulated pathways in monocytes from VEXAS versus healthy controls. **E** Detailed analysis of the two most up-regulated pathways in monocytes from VEXAS, i.e. TNF-α signaling via NF∣B pathway and hypoxia. **F** Enriched GO functions of up-regulated and down-regulated pathways in monocytes from VEXAS versus healthy controls. **G** Detailed analysis of the two most down-regulated pathways in monocytes from VEXAS, i.e. PI3K/AKT/mTOR signaling and complement pathways. **H** Top pathways enriched for pathway analysis through Wikipathways in dysregulated genes in monocytes from VEXAS versus healthy controls. **I** Expression levels in each monocyte subsets of *TYROBP*, encoding for DAP12, and *CTNNB1*, encoding catenin beta-1,in each patients’ group.

To further characterize the features of monocyte populations that characterize VEXAS blood commitment, we used single-cell RNA sequencing (scRNA-Seq) of frozen PBMCs from 2 individuals in each patient group, using the 10X Chromium droplet-based platform. Unsupervised clustering based on gene expression identified 24 cell clusters among PBMCs, depicting the classical cell types found in the blood (Supplementary Fig. 9A, B). A cluster bias analysis performed by groups of patients strikingly showed a drastic loss of clusters derived from monocytes in VEXAS patients as compared to VEXAS-like, MDS and healthy controls (Supplementary Fig. 9C, D). Interestingly, CD14^+^ CCL2^-^ and nonclassical CD14^lo^ CD16^+^ monocyte subsets were significantly reduced in VEXAS patients (Fig. 6A, B). These results confirmed major differences observed using CyTOF and showing major reduction of nonclassical CD14^lo^ CD16^+^ monocyte fractions in VEXAS patients.

When testing molecular signatures from Nanostring Human Immunology at the single-cell level on PBMCs and monocytes, high expression of IL-18, TNF-α and NFκB signatures (Fig. 6C, Supplementary Fig. 9E, Supplementary Fig. 10A) were observed as suggested by previous results (Fig. 5C), as well as a TLR4 signaling signature, known to activate and signal downstream signaling pathways to activate NFκB and inflammasome complex. We next performed differential gene expression coupled with pathways analysis enrichment and identified specific up- and down-regulated pathways in monocytes from VEXAS patients. Among the most up-regulated pathways, we identified the TNF-α signaling via NFκB pathway with a very high expression of *FOS, TNFAIP3, MAP3K8* and *KLF9* in VEXAS samples, and hypoxia (Fig. 6D, E, Supplementary Fig. 10B). Among the most down-regulated pathways, PI3K/AKT/mTOR signaling and complement pathways characterized circulating monocytes from VEXAS patients (Fig. 6F, G, Supplementary Fig. 10C).

Moreover, molecular signatures obtained from Wikipathway highlighted pathways potentially involved in the significant decrease of circulating monocytes, especially the *TYROBP* causal network in microglia (Fig. 6H). *TYROBP* and *CTNNB1* genes were downregulated in the different monocytic clusters in VEXAS patients (Fig. 6I, Supplementary Fig. 10D). Triggering Receptor Expressed on Myeloid cells 2 (TREM2), a cell surface receptor of the immunoglobulin superfamily that is expressed on microglia and myeloid cells, transmits intracellular signals through its adaptor, TYROBP/DAP12. A molecular defect of TYROBP/DAP12 in human monocytes was shown to deregulate the gene network pivotal for maintenance of myeloid cell function^21^. Also, TREM2/DAP12 signaling was shown to promote cell survival by activating the Wnt/β-catenin (*CTNNB1*) signaling pathway, at least in the central nervous system^26^ and in bone homeostasis^27^. These data cross validate finding we obtained with other approaches.

Finally, because of the upregulation of TNF-α family and NFκB signaling in whole blood and at the single-cell level, we investigated the contribution of proinflammatory programmed cell death in VEXAS. Plasma levels of lactate dehydrogenase (LDH), a marker of necrosis and cellular injury, correlated with VEXAS syndrome (Fig. 1D), as well as RIPK1 and RIPK-3, a key kinase involved in programmed necroptosis and inflammatory cell death (Supplementary Fig. 11A). Single-cell RNA sequencing of monocytes from VEXAS patients showed increased apoptosis, pyroptosis and necroptosis signatures in comparison with other groups (Supplementary Fig. 11B). IRF1, a molecule long recognized for its roles in regulating cell death, was significantly upregulated in peripheral blood of VEXAS patients (Supplementary Fig. 11C). To further confirm the involvement of necroptosis and inflammasome-mediated pyroptosis, two forms of necrotic cell death characterized by the release of the alarmin HMGB1 upon cell membrane rupture, we used a human monocytic pyroptosis and necroptosis reporter cell line (THP1-HMGB1-Lucia). We showed that the selective inhibition of ubiquitin-activating enzyme E1 with PYR-41 in combination with TNF-α stimulation induced higher HMGB1 release than PYR-41 or TNF-α alone. HMGB1 release was partially inhibited by the pan-caspase inhibitor Z-VAD-FMK and completely inhibited by the RIPK1 inhibitor necrostatin-1, supporting the major contribution of necrotic cell death in VEXAS (Supplementary Fig. 11D).

In addition, we tested molecular signatures at the single-cell level in monocytes to validate previous observations and to establish a link with impaired ubiquitination which could activate cellular stress responses and the unfolded protein response (UPR). Single-cell RNA sequencing of monocytes from VEXAS patients showed an increased UPR response and response to stress mediated by EIF2 signatures compared to other groups (Supplementary Fig. 12A). Also, detailed signatures assessing the assembly of complexes involving RIPK1, i.e. complex 1, complex 2a and complex 2b, showed upregulation of all of these pathways (Supplementary Fig. 12B). Importantly, upon activation of TNFR1, the complex 1, containing RIPK1, TNFR1-associated death domain (TRADD) and other signaling molecules, is rapidly formed and activates the induction of inflammatory and survival genes, and is subsequently followed by the assembly of complex 2a (ripoptosome) and 2b (necrosome)^27,28^.

Overall, these data confirm that monocytes from VEXAS patients display exaggerated proinflammatory signatures, UPR response and proinflammatory cell death, contrasting with a defective TYROBP/DAP12 and β-catenin signaling pathway, modulating inflammation and cell death in the peripheral blood of VEXAS.

## Discussion

By using high-throughput approaches on fresh blood samples, we were able to detail the inflammatory pathways activated in monocytes in a large cohort of VEXAS patients in comparison to adapted groups of controls, including inflammatory disorders without *UBA1* mutations. We have described unexplored areas of the myeloid/monocytic populations and have highlighted substantial differences in the abundance and phenotype of circulating monocytes in VEXAS patients. Peripheral blood monocytes from VEXAS patients showed significantly decreased counts and displayed many features of dysfunction and exhaustion, and aberrant expression of chemokine receptors. Correlation analysis of soluble mediators in peripheral blood also established that increased levels of markers of myeloid cell dysregulation and many proinflammatory cytokines such as IL-1β and IL-18 were key features of VEXAS syndrome physiopathology, confirming the potential interest of targeted therapies against these markers (IL-1β, IL1-RA, IL6 and IL-18). These data suggest that the control of the inflammasome activation could be a specific feature and a potential therapeutic vulnerability of VEXAS. It also suggests that the combination of JAKi and/or 5-azacytidine with cytokine-targeted therapies could be of interest to control inflammation in VEXAS. In vitro studies conducted on primary samples will further help us to identify the most efficient combination to control the inflammatory state observed in patients.

According to our data obtained on blood and tissues compartment, we show that the decrease of monocytes in VEXAS syndrome could be linked to an increased cell death and/or an enhanced migration into inflamed tissues. In peripheral blood, bulk RNA sequencing on immune pathways confirmed many immune dysregulations and revealed an enrichment of the TNF-α and NFκB signaling pathways. Single-cell RNA sequencing analysis of peripheral monocytes confirmed the upregulation of these pathways at the single-cell level and their association to an increased UPR signature. We also showed increased apoptosis, pyroptosis and necroptosis signatures. Necroptosis and inflammasome-mediated pyroptosis, two forms of necrotic cell death characterized by the release of the alarmin HMGB1 upon cell membrane rupture. Necroptosis and pyroptosis are respectively RIPK1 and caspase-1-dependent conditions^29–31^. Using a human monocytic pyroptosis and necroptosis reporter cell line, we observed that inhibition of UBA1 with PYR41 in combination with TNF-α induced higher HMGB1 release than PYR-41 or TNF-α alone. This release was partially inhibited by the pan-caspase inhibitor Z-VAD-FMK and almost totally inhibited by the RIPK1 inhibitor necrostatin-1 suggesting the induction of necrotic cell death. Further studies allowing the analysis of neutrophils from fresh blood samples will be useful to add important information on the pathophysiology of this disorder.

The poor efficacy of conventional disease-modifying antirheumatic drugs to control VEXAS could support the concomitant engagement of different modes of programmed cell death^31^. Our high-throughput analyses also highlighted a defective TYROBP/DAP12 and β-catenin signaling pathway in circulating monocytes which could explain a defect in survival signals. As mentioned, TREM-2 is expressed on myeloid cells and transmits intracellular signals through its adaptor, TYROBP/DAP12 and a molecular defect in TYROBP/DAP12 of human monocytes was shown to deregulate a gene network critical for maintenance of myeloid cell function. TREM-2/DAP12 signaling was also shown to promote cell survival by activating the Wnt/β-catenin (*CTNNB1*) signaling pathway in the central nervous system and in the bone. Thus, dysregulation of the TYROBP/DAP12 pathway may be involved in the monocytopenia and increased inflammatory cell death observed in VEXAS patients. However, regulation of TREM-2 and TYROBP expression and the transcription factors required for their expression are largely unknown. Further studies could investigate whether *UBA1* mutation affects the expression of TREM-2 and TYROBP. Future work will be also needed to determine whether targeting cell death can be used as a therapeutic strategy in VEXAS. A monitoring of these dysregulations would be investigated in vivo and in vitro to appreciate the efficacy of treatments.

A strength of this study is the possibility to obtain information about the fate of myeloid cells in tissues that seems different to their behavior in peripheral blood. As expected, bone marrow analysis shows an overrepresentation of the myeloid cell lineage with mature and immature forms which contrasts with observations in peripheral blood. Dysregulation of the bone marrow microenvironment was showed in the pathophysiology of many myeloid disorders by influencing the behavior of the stem cell compartment^32^. The role of the bone marrow microenvironment in VEXAS syndrome is currently unknown and its exploration should be an important field of investigation especially in terms of proinflammatory molecules secretion by stromal cells and survival factors for mutated cells. In the skin of VEXAS patients, we showed abundant monocytes and macrophages. High expression of chemokine receptors by circulating monocytes supported by CyTOF data and increased levels of chemokines, especially the CCL2/CCR2 axis, also support an enhanced migration of monocytes into tissues. Future studies will be necessary to identify the signals which allow the skin infiltration by mutated cells and to determine the potential protective role of this environment. If specific pathways are involved in this propagation of the mutated cells, some therapeutic options should be considered in addition to current treatments.

Our study has however some limitations. The difficulty to isolate sufficient circulating monocytes from VEXAS patients did not allow us so far to evaluate more precisely the mechanisms driving their important decrease, especially regarding cell death pathways. Also, no viable mice or cell-based model allowing the analysis of functional consequence of *UBA1* mutations currently exist, so we were not able to study the role of TYROBP/DAP12 and β-catenin signaling pathway. Future experiments on sorted monocytes from VEXAS and controls, on genetically modified monocytic cell lines or on bone marrow-derived monocytes will be needed to provide data on the biology of mutated cells. Another limitation of our work was associated with the scRNA-Seq approach. We try to evaluate the presence of single nucleotide variation in both UBA1 and DNMT3A VEXAS patients using cb_sniffer (https://github.com/sridnona/cb_sniffer). This tool retrieves the gene specific reads in each sample and classifies a cell as “mutant” if it contains at least one variant read. We ran cb_sniffer with defaul parameters and the list of variants expected in the two VEXAS patients. Even when focusing on the specific position of the UBA1 and DNMT3a mutations described in the 2 VEXAS patients, our analyses are inconclusive most probably due to the low coverage and sequencing depth observed at those regions, combined with the very low number of monocytes left in those patients. Once again as our experiments were not design to find such mutations it remains difficult to draw any conclusions from our single-cell datasets. Finally, further studies including more scRNA-Seq data sets will be helpful to validate these finding which are cross validated by other technics we used.

Although future works and clinical trials targeting cell death and inflammasome pathways in VEXAS have yet to be completed, based on our observations, such pathways could be highlighted as therapeutic possibilities. The role of a residual normal hematopoiesis in VEXAS patients and the evolution of the circulating monocytes under treatments will also represent major points to monitor in prospective clinical trials. Moreover, in a near future, these approaches will be used to study VEXAS-like disorders whose genetic background is not yet elucidated, to patients with atypical *UBA1* mutations as recently reported^33,34^ and to extend these approaches to a larger cohort of MDS.

## Online content

Any methods, additional references, reporting summaries, source data, extended data, supplementary information, acknowledgements, peer review information; details of author contributions and competing interests; and statements of data and code availability are available online. GSE216548 contains all the files required to reproduce our analyses (FastQ and the processed data from Cell ranger, plus the integration (rds object from Seurat)

## Methods

No statistical methods were used to predetermine sample size. The experiments were not randomized, and the investigators were not blinded to allocation during experiments and outcome assessment. Our research complies with all relevant ethical regulation, as detailed in the ‘Cohorts’ section.

### Cohort

This non-interventional study was conducted between January, 2021 and May, 2021, in the setting of the local RADIPEM biological samples collection derived from samples collected in routine care. Biological collection and informed consent were approved by the Direction de la Recherche Clinique et Innovation (DRCI) and the French Ministry of Research (No. 2019-3677). A written informed consent was collected for all participants. None of the study participants received compensation. This study was conducted in compliance with the Good Clinical Practice protocol and the principles of the Declaration of Helsinki, and received approval from the Cochin Hospital Institutional Review Board (number AAA-2021-08040).

Patients included in this study were: adults patients aged over 18 years old, with a history of inflammatory syndrome characterized by relapsing polychondritis, Sweet’s syndrome, polyarteritis nodosa, or other unclassified inflammatory disease, and associated or not with well-defined hematologic condition (mainly myelodysplastic syndrome). Epidemiological, demographic, clinical, laboratory, treatment, and outcome data were extracted from medical charts using a standardized data collection form. At time of sampling and according to the pandemic situation, all included patients were tested for SARS-CoV-2 infection and were all negative.

All the blood samples selected for this study were received and used 24 h after blood collection.

For VEXAS, VEXAS-like and MDS patients, genomic DNA was extracted from blood samples and the third exon of the gene was first analyzed by Sanger approach to detect the described mutations using specific primers as previously reported (Forward 5′-TCCAAAGCCGGGTTCTAACT-3′/Reverse 5′-GGGTGTGCAGTAGGGAAAAA-3′)^2^. We also performed a NGS characterization of the whole cohort, as previously reported^34^ with an updated panel incorporating the UBA1 full gene sequence (NM_003334.4). Results are presented in the Suppl Fig. 1 and allow the description of 2 patients harboring subclonal mutations of UBA1, with 9 and 6% of VAF respectively. No other mutation of UBA was identified in this cohort. The molecular myeloid landscape of the VEXAS patients fit with the recent description^10^.

### Routine plasmatic biomarkers measurements

Routine blood examinations were complete blood count and plasmatic biochemical tests, including C-reactive protein (CRP), ferritin, interleukin-(IL)−6, calprotectin and lactate dehydrogenase (LDH). Plasma EDTA were collected and frozen at −80 °C for subsequent measurement of protein biomarkers. Lactate-deshydrogenase (LDH) concentrations were measured using the LDHI2 enzymatic UV assay on a cobas c701 analyzer (Roche Diagnostics Meylan, France). The measuring range of the assay extended from 10 to 1000 UI/L. CRP concentrations were measured using the Tina-quant CRP-Gen3 immunoturbidimetric assay on a cobas c701 analyzer (Roche Diagnostics Meylan, France). The measuring range of the assay extended from 0.5 to 15 mg/L. Physicians in charge of the patients were blinded to the results of biomarkers, and biologists were blinded to the emergency diagnosis suspected by physicians.

### Multiparameter phenotyping of peripheral blood leukocytes using mass cytometry

The Maxpar® Direct™ Immune Profiling System (Fluidigm, Inc Canada) was used for high-dimensional immune profiling of whole blood, using a 30-marker antibody panel with the addition of eleven markers: anti-CD11b conjugated to 106 Cd, anti-CD64 conjugated to 111 Cd, anti-CCR3 conjugated to 113 Cd, anti-FcERI conjugated to 116 Cd, anti-CD69 conjugated to 142Nd, anti-CD117 conjugated to 165Ho, anti-CD336/NKp44 conjugated to 169Tm, anti-Tim3 conjugated to 159Tb (1 μg/μL concentration), anti-CD335/NKp46 conjugated to 162Dy (1 μg/μL concentration), anti-PD-1 conjugated to 175Lu (1 μg/μL concentration) and anti-PD-L1 conjugated to 209Bi (1 μg/μL concentration). For each sample, 10 μL of 10 KU/mL heparin solution was added to 1 mL of whole blood, then the samples were incubated for 20 min at room temperature. After incubation, 270 μL of heparin treated whole blood was directly added to the dry antibody cocktail and additional antibodies were added, for an incubation of 30 min. Red blood cells were then lysed immediately using CAL-Lyse Lysing Solution (Life Technologies). After a 10-min incubation, 3 mL of MaxPar Water was added to each tube for an additional 10-min incubation. Cells were washed three times using MaxPar Cell Staining Buffer and then fixed for 10 min using with 1.6% paraformaldehyde (Sigma-Aldrich, Lyon, France). Cells were washed once with MaxPar Cell Staining Buffer and incubated one hour in Fix and Perm Buffer with 1:1000 of Iridium intercalator (pentamethylcyclopentadienyl‐Ir (III)‐dipyridophenazine, Fluidigm, Inc Canada). Cells were washed and resuspended in Maxpar Cell Acquisition Solution, a high-ionic-strength solution, at a concentration of 1 million cells per mL and mixed with 10% of EQ Beads immediately before acquisition. Cell events were acquired on the Helios mass cytometer and CyTOF software version 6.7.1014 (Fluidigm, Inc Canada) at the “Plateforme de Cytométrie de la Pitié‐Salpetriere (CyPS).” An average of 400,000 events were acquired per sample. Mass cytometry standard files produced by the HELIOS were normalized using the CyTOF Software v. 6.7.1014. This method normalizes the data to a global standard determined for each log of EQ beads.

FCS3.0 files generated by the Helios were analyzed using GemStone software (Verity Software House, Topsham, ME), an automated analysis system. This system is integrated with dimensionality‐reduction mapping known as Cauchy Enhanced Nearest‐neighbor Stochastic Embedding (Cen-se™), which generates a visual display of high‐dimensional data labeled with the major cell populations.

The multiparametric analysis of activation and immune checkpoint markers was performed on FlowJo and the data generated were then analyzed using Tableau Desktop. For viSNE analysis (Cytobank Inc, Mountain View, CA, USA), mapping integrating a total of 50,000 PBMCs sampled and analyzed for each group FCS file. Parameter viSNE maps were created with all markers. The settings used for the viSNE run were as follow: iterations (3000), perplexity (70) and theta (0.5). ViSNE maps are presented as means of all samples in each disease severity category.

### Imaging mass cytometry

Tissue sections were dewaxed in xylene for 20 min. Samples were rehydrated in a graded series of alcohol (ethanol:deionized water 100:0, 95:5 and 70:30; 5 min each). After that, slides were washed in Maxpar Water® for 5 min in a Coplin jar placed on an orbital shaker plate with gentle agitation. Slides were put into to Tris-EDTA buffer at pH 8 followed by heat-induced epitope retrieval in a pressure cooker (Medite) for 25 min at 92 °C. Samples were left to cool in the Tris-EDTA buffer for 20 min followed by cooling in Tris-buffered saline (TBS) at room temperature for at least 20 min.

After that, slides were washed in Maxpar Water® for 10 min in a Coplin jar placed on an orbital shaker plate with gentle agitation, then in Maxpar PBS® for 10 min. Slides were then blocked with blocking buffer containing 3% BSA in Maxpar PBS® for 45 min. Samples were then stained with antibody mix (diluted in blocking buffer) and incubated overnight at 4 °C. Samples were washed three times in 0.2% Triton X-100 in Maxpar PBS® for 8 min with slow agitation in Coplin jars, then in Maxpar PBS® for 5–10 min, before incubating for 30 min with iridium intercalator diluted in Maxpar PBS® followed by three washes in Maxpar PBS® (5 min each wash). Samples were then air dried before IMC acquisition.

Data acquisition was performed on a Helios time-of-flight mass cytometer (CyTOF) coupled to a Hyperion Imaging System (Fluidigm). Selected areas for ablation were larger than the actual area of interest to account for loss of overlapping areas among sections due to cumulative rotation. The selected area ablated per section was around 1 mm^2^. To ensure performance stability, the machine was calibrated daily with a tuning slide spiked with five metal elements (Fluidigm). All data were collected using the commercial Fluidigm CyTOF software v.01. Finally, regions of interest with markers of interest (DNA1, smooth muscle actin [SMA], CD3, CD4, CD8, CD14, CD16, CD31, CD68 and CD163) were visualized using MCD^TM^ Viewer v1.0.560.6 software.

For each sample, genomic DNA was extracted and the mutational status of UBA1 was tested as previously reported. The VEXAS skin biopsies presented the same UBA1 mutation that was identified in fresh blood samples.

### Cytokine assays

Plasma were analyzed using the Luminex xMAP technology and a 45 plex assay (R&D Systems). To quantify IFN-α, IFN-γ and IL-17A at ultrasensitive concentrations, Single Molecule array (Simoa) technology was used and a homebrew triplex assay was developed as previously described (Bondet et al, 2021). All the assays were run on a 2-step configuration on the Simoa HD-1 Analyzer (Quanterix, US). Limit of detection (LoD) was defined (using the highest bottom value of 95% CI in Prism 9). The R package “limma” v3.44.3 was used to correct for Luminex kits batch effect. PCA and violin plots were performed using the R packages “factoextra” v1.0.7 and “ggplot2” v3.3.3 respectively. Interleukin-6 concentrations were measured using the IL-6 ECLIA assay on a cobas E801 analyzer (Roche Diagnostics Meylan, France). The measuring range of the assay extended from 0.5 to 5000 ng/L. Calprotectin concentrations were measured using the turbidimetric assay (Gentiane) on a cobas c501 analyzer (Roche Diagnostics Meylan, France). The measuring range of the assay extended from 0.5 to 15 mg/L. All quality controls during the study were conformed to. IL-1β (Human IL-1 beta/IL-1F2 Quantikine ELISA Kit, Catalog # DLB50, R&D Systems), IL-18 (Human Total IL-18/IL-1F4 Quantikine ELISA Kit, Catalog # DL180, R&D Systems) and galectin-3 (Human Galectine-3 Quantikine ELISA Kit, Catalog # DGAL30, R&D Systems) plasma levels were measured using an ELISA kit and following the manufacturer’s instructions.

### Whole blood stimulation with crystals and LPS

Fresh blood was collected from VEXAS, VEXAS-like and gout patients. A total of 200 μL of fresh blood was cultured in 800 μL serum-free media with antibiotics and then stimulated with PBS, MSU, m-CPPD crystals (200 µg/ml) and LPS (10 ng/ml, Sigma) for 24 hrs. Whole blood culture were collected spined to collect the supernatant and stored at −20 °C. IL-1β levels in supernatants were measured using an ELISA kit and following the manufacturer’s instructions (Invitrogen 88-7261-88).

### Pyroptosis and necroptosis cell line assay

The human THP-1 monocytic cell line (THP-1-HMGB1-Lucia, InvivoGen, San Diego, CA, USA) was used to assess pyroptosis and necroptosis. THP1-HMGB1-Lucia cells secrete recombinant HMGB1::Lucia luciferase fusion protein when the cells undergo pyroptosis and/or necroptosis. THP-1 cells were incubated in a T-75 flask containing Roswell Park Memorial Institute 1640 Medium GlutaMAX™ Supplement medium (RPMI 1640 GlutaMAX™; Gibco, Grand Island, NY, USA) supplemented with 25 mM HEPES, 10% heat-inactivated fetal bovine serum (FBS), 100 U/mL penicillin, 100 μg/mL streptomycin, 100 µg/mL of Normocin (InvivoGen), and 100 µg/mL of Zeocin (InvivoGen) at 37 °C, in a humidified 5% CO_2_ incubator according to the supplier’s instructions. For HMGB1 release measurement, THP-1 cells were cultured at 1 × 10^6^ cells/mL in 1 mL RPMI 1640 GlutaMAX supplemented medium. Cells were pre-treated with a selective inhibitor of ubiquitin-activating enzyme E1 PYR-41 (5 µM) with or without the pan-caspase inhibitor Z-VAD-FMK (20 μM) or RIPK1 inhibitor Necrostatin-1 (30 µM) for 1 h prior to incubation with recombinant human TNF-α (50 ng/ml). After 24 h, the luciferase activity was determined by measuring relative light units (RLUs) in a luminometer using QUANTI-Luc™ detection reagent (Normocin: InvivoGen, catalog code: ant-nr-05; Zeocin: InvivoGen, catalog code: ant-zn-1p; PYR-41: TargetMol, catalog code: T6629; TNF-α: Bio-Techne, catalog code: 210-TA; Necrostatin-1: InvivoGen, catalog code: inh-ncst1; Z-VAD-FMK: InvivoGen, catalog code: d-tlrl and QUANTI-Luc: InvivoGen, catalog code: rep-qlc1).

### Gene expression analysis

Total RNA was extracted from 2 ml of whole blood collected on EDTA using the Promega Maxwell LEV Simply RNA Kit (Ref AS1280). RNA concentrations were measured using a Nanodrop One (Thermo Scientifics). Total RNA samples were analyzed using the Human Immunology kit v2 panel profiling 594 immunology-related human genes according to manufacturer’s instructions (Nanostring). Gene expression data were normalized using control probes and housekeeping genes selected using the geNorm method of the Advanced Analysis package (nSolver Analysis Software 4.0). As gene expression was measured using two different batches of CodeSets, batch effect correction was performed using the removeBatchEffect function of the R package “limma” v3.44.3.

To identify gene expression differences between groups, multiple linear regression was performed while correcting for lymphocyte, neutrophil and monocyte cell proportions to control for potential gene expression differences due to cellular differences between patients. The analysis was implemented using the R package “broom”‘ v0.7.5. Multiple testing correction was then applied to select the significant genes. PCA, volcano plots and heatmaps were performed using the “factoextra” v1.0.7, “EnhancedVolcano” v1.6.0 and “pheatmap” v1.0.12 respectively. Finally, gene signatures (Supplementary table 3) were calculated for each sample by using the zScore function of the R package multiGSEA v1.1.99 and gene set enrichment analysis was performed as previously described^35^.

Total skin RNA was extracted from lesional and non lesional skin using Trizol (Invitrogen) and RNeasy mini kit (Qiagen) in accordance with the manufacturers’ instructions. RNA sequencing was performed at the Genomic Platform of Institut Cochin, with an Illumina NextSeq 500 device (2 reads x 25 million fragments, paired-end 2 × 75 nt). Quantification via pseudoalignement was performed using Kallisto version 0.46.1 to estimate abundances of transcripts using the Ensembl homo sapiens transcriptomes v96 as an index^36^. Pseudoalignment count tables in TPM (transcripts per million) were used. Analysis was performed using a custom R script using the following R packages: tximeta to summarize transcript-level quantification to the gene-level and the caroline package to compute data for RA-plots using the raPlot function^37^. Data were visualized using Ingenuity Pathway Analysis (Qiagen). Comparison between lesional and normal skin was performed using the exact test for differences between two groups of negative-binomial counts from the EdgeR package using 0.4 as an estimate of the biological coefficient of variation. *p*-values are adjusted for multiple comparisons using the Benjamini & Hochberg method.

### Single-cell RNA sequencing

In brief, thawed PBMCs were used to perform the scRNA-Seq experiment. The scRNA-seq libraries were generated using Chromium Single Cell Next GEM 3′ Library & Gel Bead Kit v.3.1 (10′ Genomics) according to the manufacturer’s protocol. Briefly, defrozen PBMCs were counted, diluted at 1000 cells/µL in PBS and 20,000 cells were loaded in the 10 × Chromium Controller to generate single-cell gel-beads in emulsion. After reverse transcription, gel-beads in emulsion were disrupted. Barcoded complementary DNA was isolated and amplified by PCR. Following fragmentation, end repair and A-tailing, sample indexes were added during index PCR. The purified libraries were sequenced on a Novaseq (Illumina) with 28 cycles of read 1, 8 cycles of i7 index and 91 cycles of read 2.

Sequencing reads were demultiplexed and aligned to the human reference transcriptome (GRCh38-2020-A directly download from 10 ×), using the CellRanger Pipeline (v-5.0.1).

The unfiltered raw UMI counts from cellranger were loaded inot Seurat v4.0.3 (Stuart et al., 2019) for quality control, data integration and downstream analyses. Doublets, empty sequencing beads and apoptotic cells were removed by filtering out cells with fewer than 500 features or a mitochondrial content higher than 20%. Quality controls of cells selected for further analysis are summarized in Supplementary Table 6. Data from each sample were normalized and scaled using the sctransform method, and batch effect between samples was corrected using Seurat’s FindIntegratedAnchors.

On this integrated dataset, we computed the principal component analysis on the 2000 most variable genes. UMAP was carried out using the 20 most significant PCs, and community detection was performed using the graph-based modularity-optimization Louvain algorithm from Seurat’s FindClusters function with a 0.8 resolution.

Cell type labels were assigned to resulting clusters based on a manually curated list of marker genes as well as previously defined signatures of the well-known PBMC subtypes (Monaco et al., 2019). All clusters were annotated, and 63630 cells were kept for further analysis.

Differential expression was performed on different groups, using the FindMarkers function of Seurat on the RNA assay with default parameters (Wilcoxon testing with Bonferroni correction). Only genes with adjusted *p*-values < 0.05 were selected as significant. The lists of differentially expressed genes were further divided into UP and DOWN regulated genes based on the avg_log2FC; avg_log2FC > 0 for the UP regulated genes and and avg_log2FC < 0 for the DOWN regulated ones.

The signature scores were calculated using the function AddModuleScore from Seurat, and dot plots were used to visualize the change in signature signal between conditions.

The data for the analysis on the myeloid and monocyte populations were obtained using the subset function from Seurat.

### Statistical analysis

CyTOF data were analyzed with FlowJo version 10 software. Calculations were performed using Excel 365 (Microsoft). Figures were drawn on Prism 9 (GraphPad Software). Statistical analysis was conducted using GraphPad Prism 9. *P* values were determined by a Kruskal-Wallis test, followed by Dunn’s post-test for multiple group comparisons with median reported; *P < 0.05; **P < 0.01; ***P < 0.001. All tests were two-sided. Correlation matrices of cytokines from patient blood were calculated. Only significant correlations (<0.05) are represented in color.

### Statistics and reproducibility

All samples discussed in this manuscript were measured and analyzed as technical duplicates, except for the Simoa and the Luminex data that were analyzed as technical simplicates.

### Reporting summary

Further information on research design is available in the Nature Portfolio Reporting Summary linked to this article.

### Supplementary information

Supplementary Information

Description of Additional Supplementary Files

Supplementary Data 1

Supplementary Data 2

Reporting Summary

### Source data

Source Data

## Data Availability

All data supporting the findings of this study are available within the article or from the corresponding authors upon reasonable request without any restrictions. GSE216548 contains all the files required to reproduce our analyses (FastQ and the processed data from Cell ranger, plus the integration (rds object from Seurat) https://www.ncbi.nlm.nih.gov/geo/query/acc.cgi?acc=GSE216548 Source data are provided with this paper.
