# Supplementary material for: VEXAS syndrome is characterized by inflammasome activation and monocyte dysregulation
Source: Nat Commun. 2024 Jan 30;15:910. doi: 10.1038/s41467-024-44811-4 (PMC10828464; doi:10.1038/s41467-024-44811-4)
Supplement: Supplementary file 1 — Supplementary Information [file 41467_2024_44811_MOESM1_ESM.pdf]

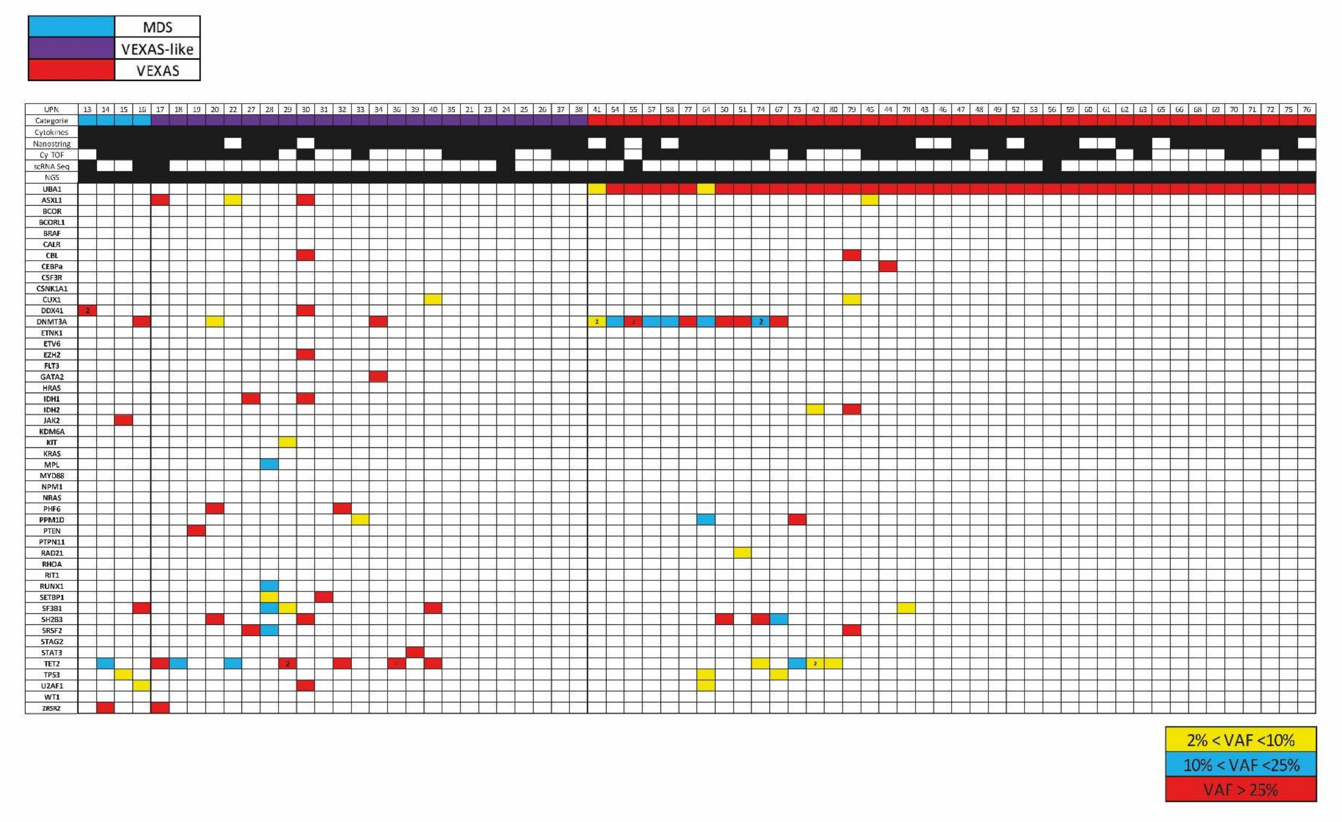

Supplementary Fig. 1. Summary of OMICS analysis performed and the presence of genomic mutations present in all patients analyzed. An NGS analysis on genomic DNA was performed as previously reported (Papo et al) with a modified panel including the UBA1 full gene sequence (NM_003334.4). Among the 48 genes tested, mutations were found in 30 genes and indicated according to the VAF. All the 40 VEXAS patients present an UBA1 mutation with only two patients with low VAF and with at least one additional mutation in 18 cases. Four patients present 2 mutations of the same gene indicated by a "2" in the case (3 cases of DNMT3A and 1 case of TET2).

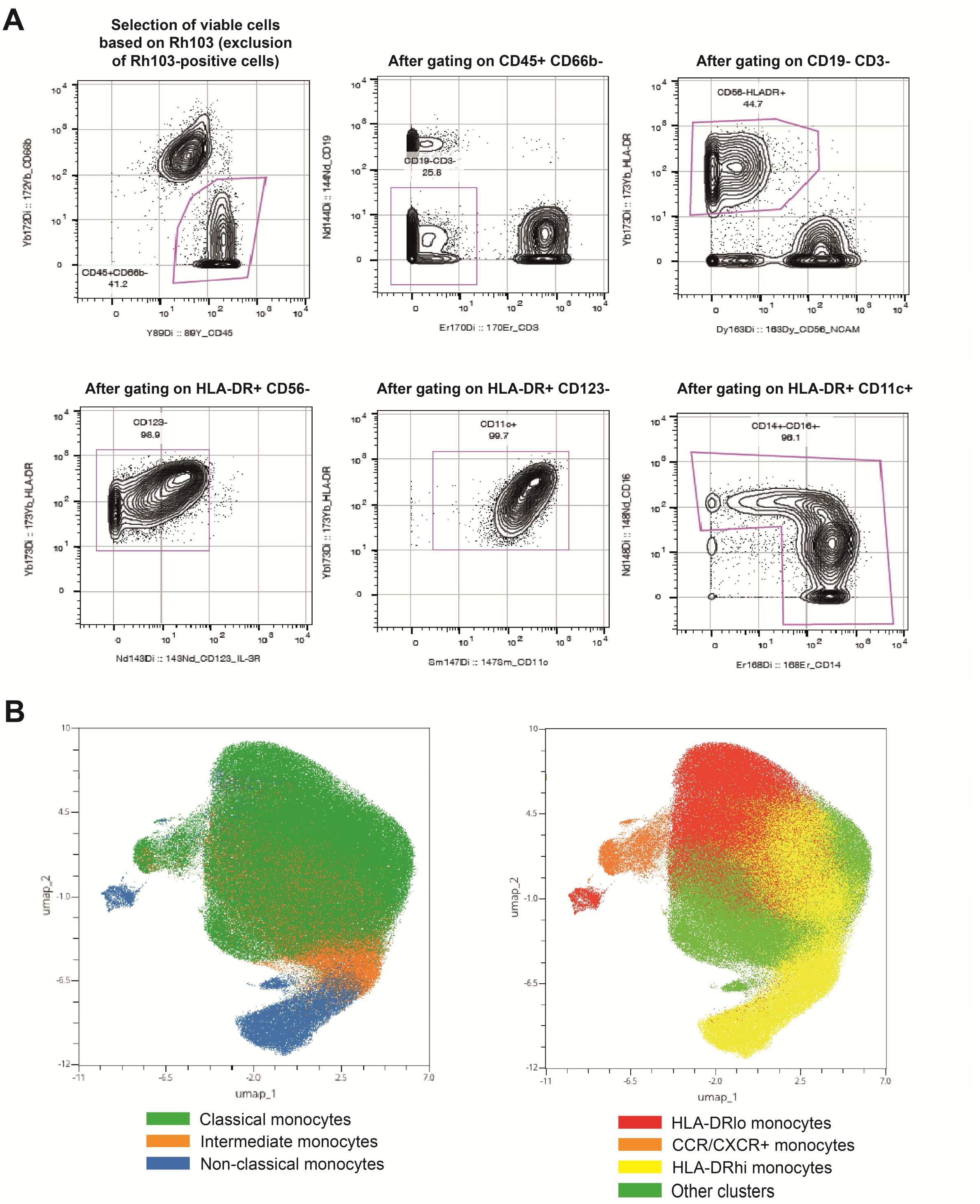

Supplementary Fig. 2. Additional information on mass cytometry of patients. (**A**) Gating strategy used for the analysis of monocytes. **(B)** Non-supervised Uniform Manifold Approximation and Projection (UMAP) of blood monocytes according to surface markers and main monocytes subsets.

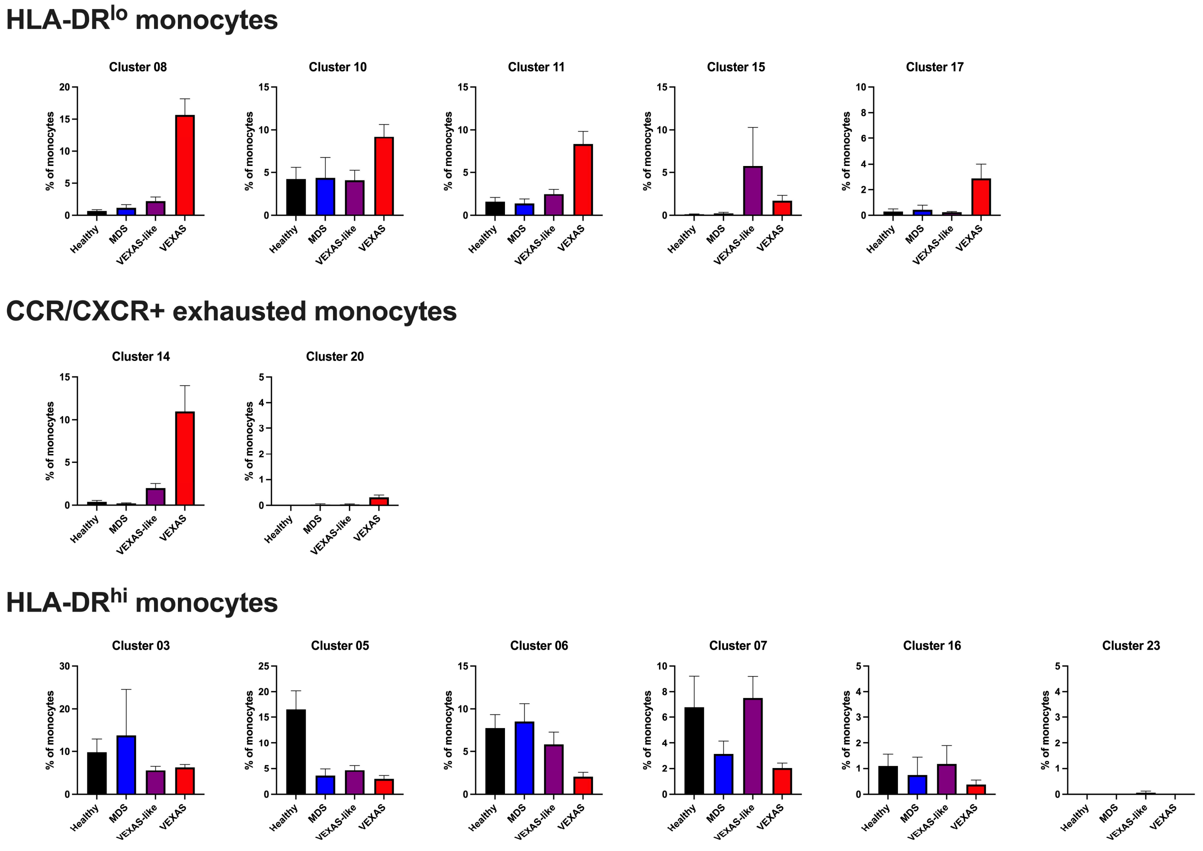

Supplementary Fig. 3. Distribution of monocyte clusters among whole monocytes in patients with VEXAS syndrome, VEXAS-like, MDS and healthy controls.
Clusters 8, 10, 11, 15 and 17, corresponding to HLA-DR^{lo} monocytes (detailed in upper panel), and clusters 14 and 20 (middle panel), corresponding to exhausted monocytes expressing chemokine receptors, are increased in VEXAS. Clusters 3, 5, 6, 7 and 16 (lower panel), corresponding to HLA-DR^{hi} monocytes, are decreased in VEXAS.

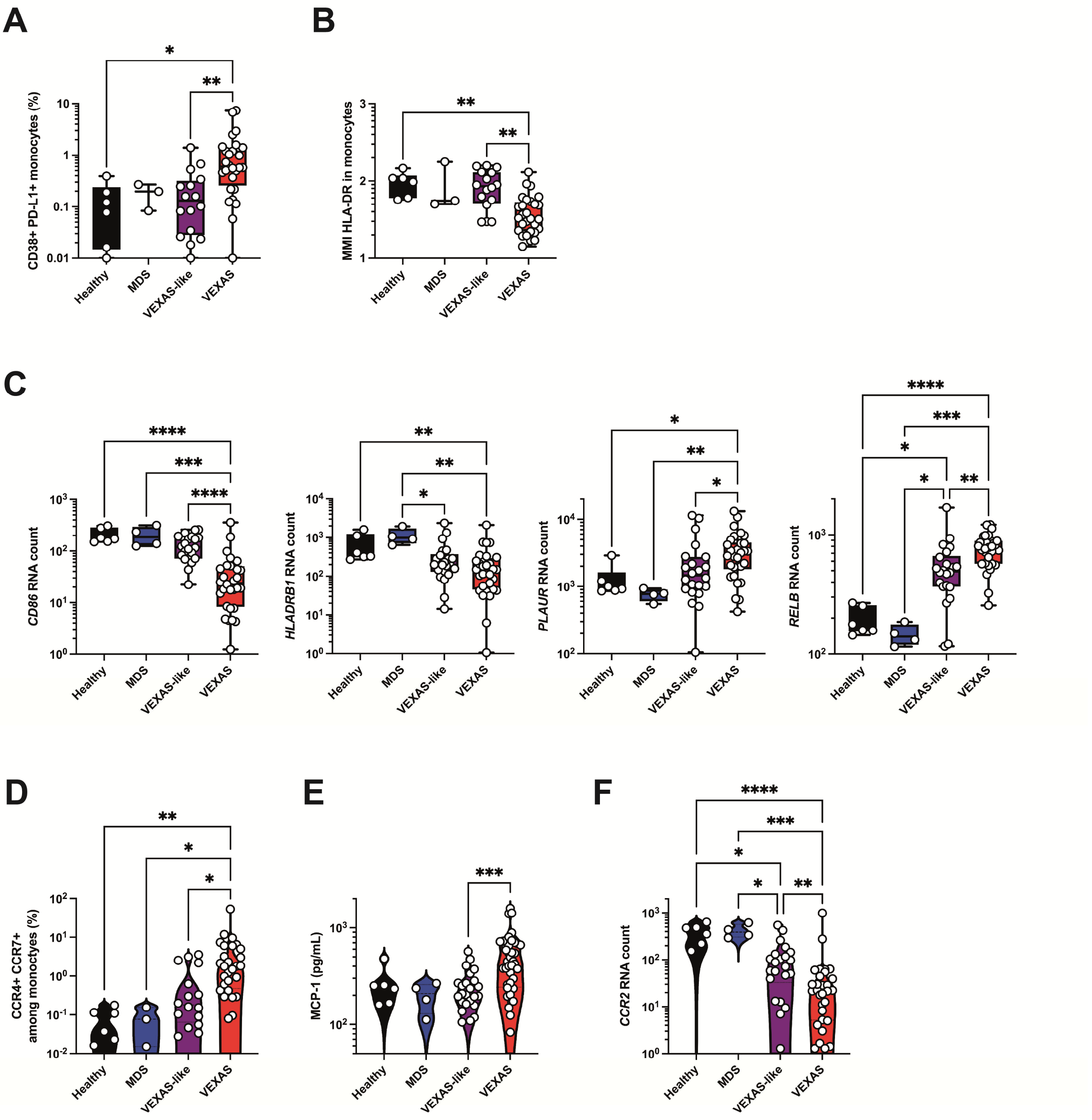

Supplementary Fig. 4. Activation status of monocytes at cellular and molecular level. Each dot represents a single patient studied as described by CyTOF, cytokines measurement or RNA studied by Nanostring nCounter analysis. **(A)** Analysis of exhausted monocytes based on the expression of CD38 and PD-L1 by mass cytometry. Each dot represents a single patient. **(B)** Cell surface expression of HLA-DR on monocytes, evaluated by mean metal intensity (MMI). **(C)** Absolute RNA counts using Nanostring nCounter technology and showing decreased expression of CD86 and HLADRB1, and increased expression of PLAUR and RELB transcripts in VEXAS in comparison to other groups. **(D)** Proportion (frequencies) of CCR4+ CCR7+ expressing monocytes. **(E)** CCL2/MCP-1 protein plasma concentration measured with Luminex technology, and **(F)** absolute RNA count for CCR2. Each dot represents a single patient. RNA data are extracted from the Nanostring nCounter analysis. P values were determined by the two-sided Kruskal-Wallis test, followed by Dunn's post test for multiple group comparisons. *P <0.05; **P <0.01; ***P <0.001, ****P <0.0001.

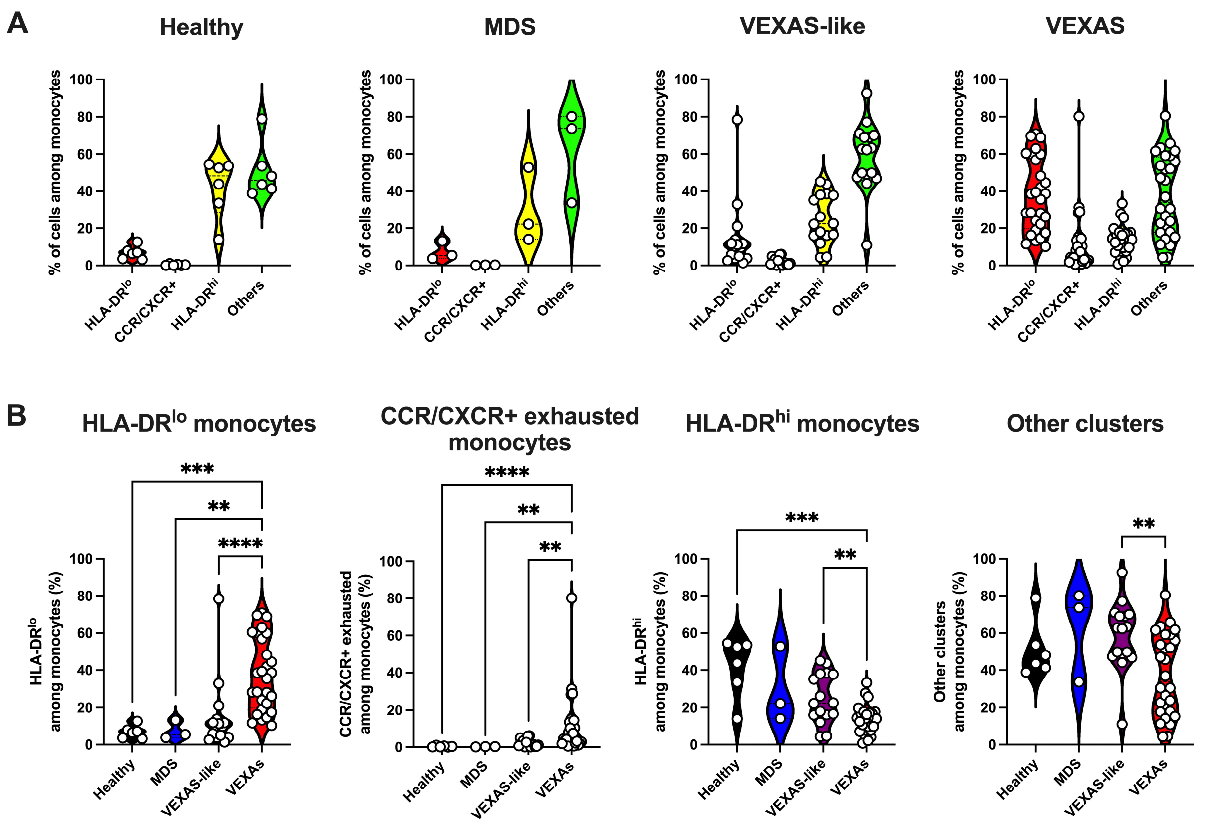

Supplementary Fig. 5. Expression of cell markers by individual representation of the phenotypic clusters in each patient group. (A) Representation of different monocytes clusters in each group of samples. (B) Individual representation of patients in each cluster allowing visualization of individual values from each patient group

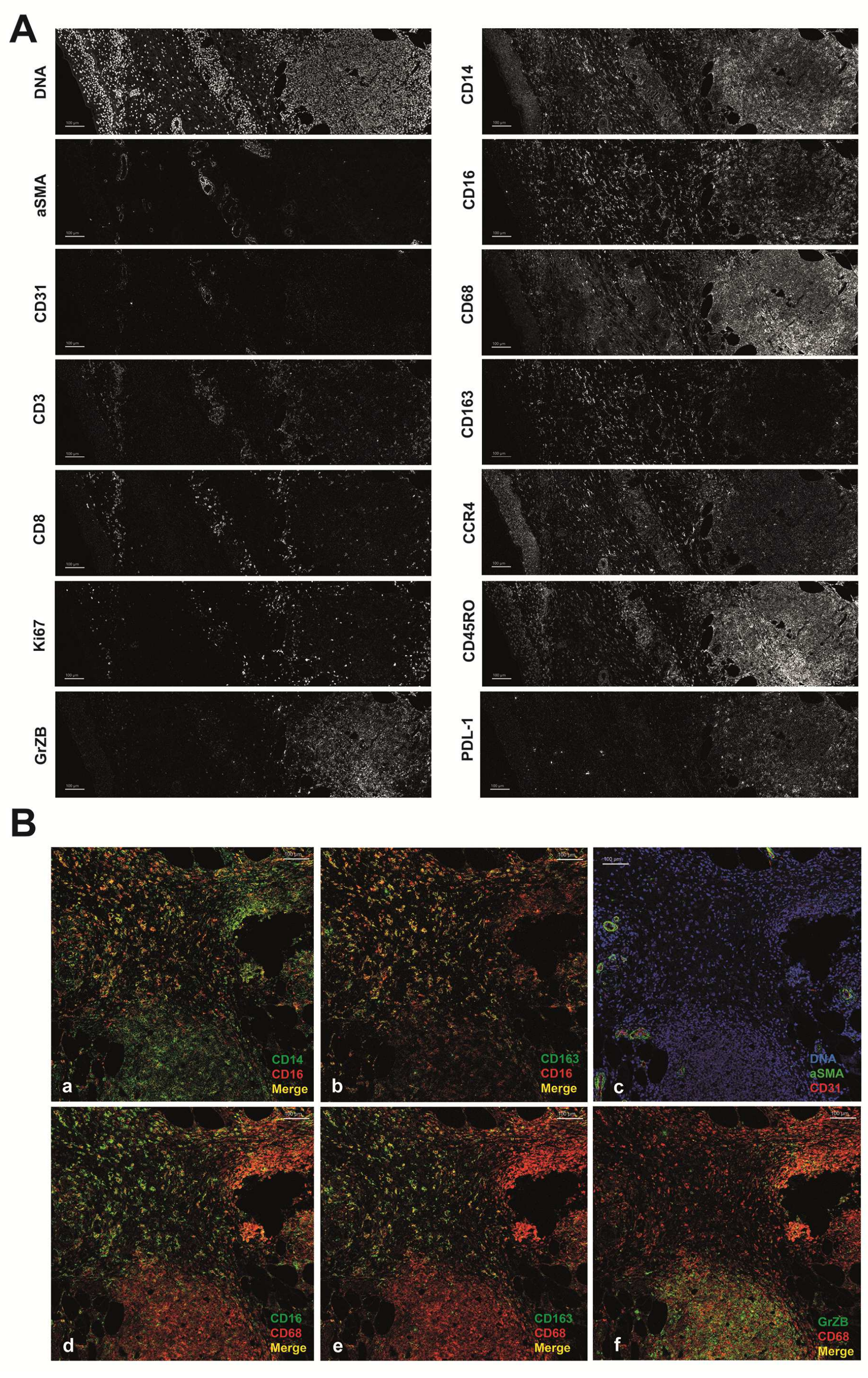

Supplementary Fig.6. Expression of cell markers on pathological skin lesion from a VEXAS patient using imaging mass cytometry.

(A) Imaging mass cytometry on skin biopsy showing expression of DNA, alpha-smooth muscle actin (aSMA), CD31, CD3, CD8, CD14, CD16, CD68, CD163, CCR4, Ki67, CD45RO, granzyme B (GrZB) and PDL-1. (B) Imaging mass cytometry on skin biopsy showing abundant nonclassical ($CD14^{lo}CD16^{+}$) and intermediate ($CD14^{+}CD16^{+}$) monocytes expressing CD163, adjacent to blood vessels, and $CD68^{+}CD163^{-}$ M1 macrophages forming clusters away from blood vessels. Illustrative pictures are shown.

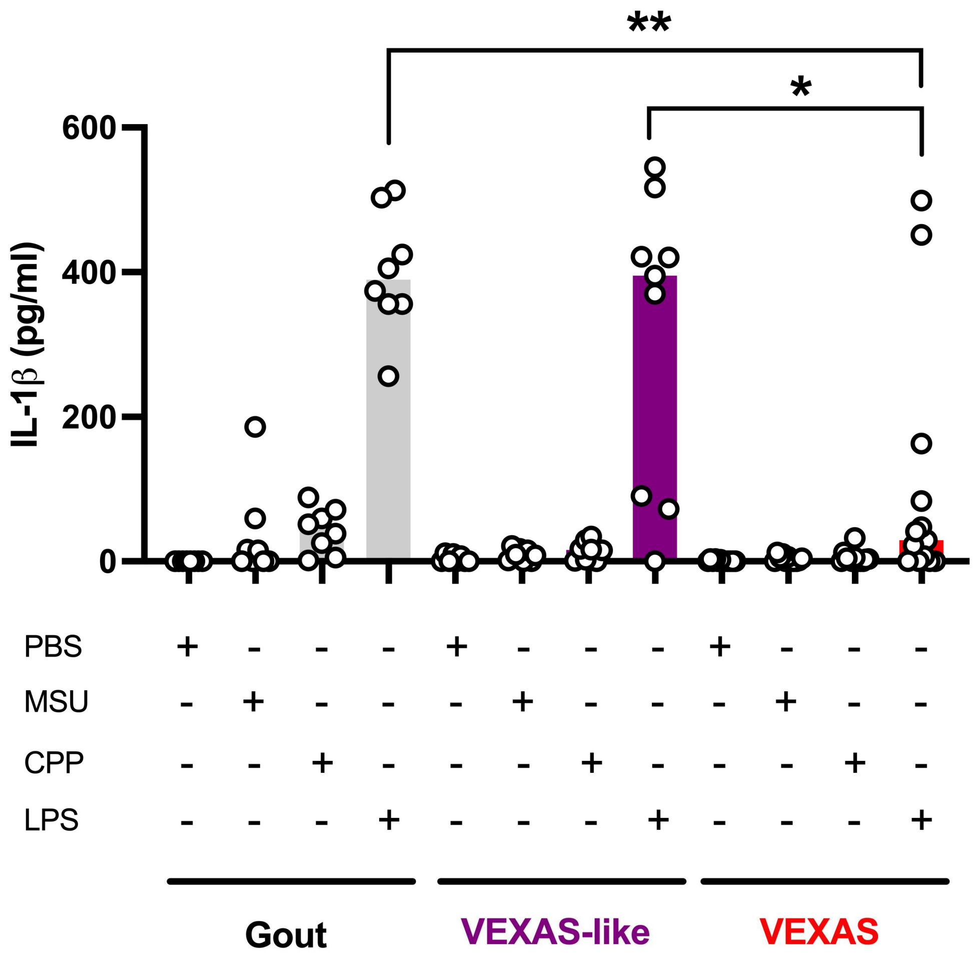

Supplementary Fig. 7. Whole blood stimulation with crystals and LPS from patients with VEXAS, VEXAS-like and gout.
Fresh blood were collected from VEXAS, VEXAS-like and gout patients and stimulated with PBS, monosodium urate (MSU), monoclinic calcium pyrophosphate dihydrate (m-CPPD) crystals (200 µg/ml) and LPS (10 ng/ml) for 24 hrs. IL-1β levels in supernatants were measured using ELISA.

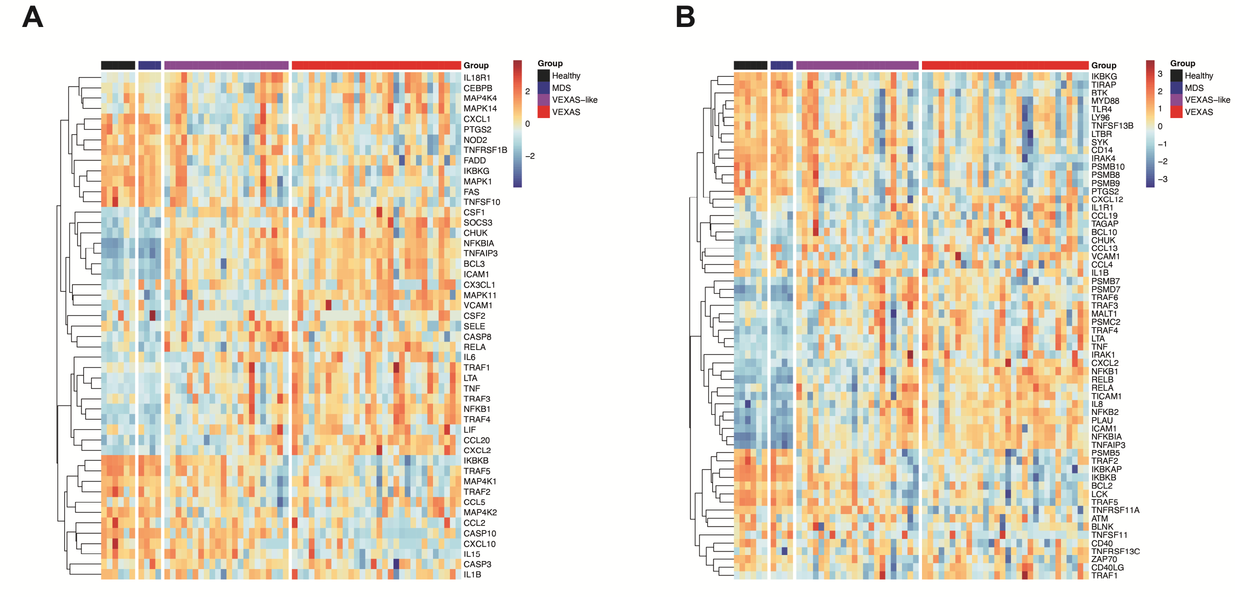

Supplementary Fig. 8. TNF-α pathway signaling and NF|B signaling signatures in patients with VEXAS syndrome, VEXAS-like, MDS and healthy controls.
(A) Heatmap representation of genes encoding proteins involved in TNF-α pathway signaling in each group. **(B)** Heatmap representation of genes encoding proteins involved in NFκB signaling in each group.

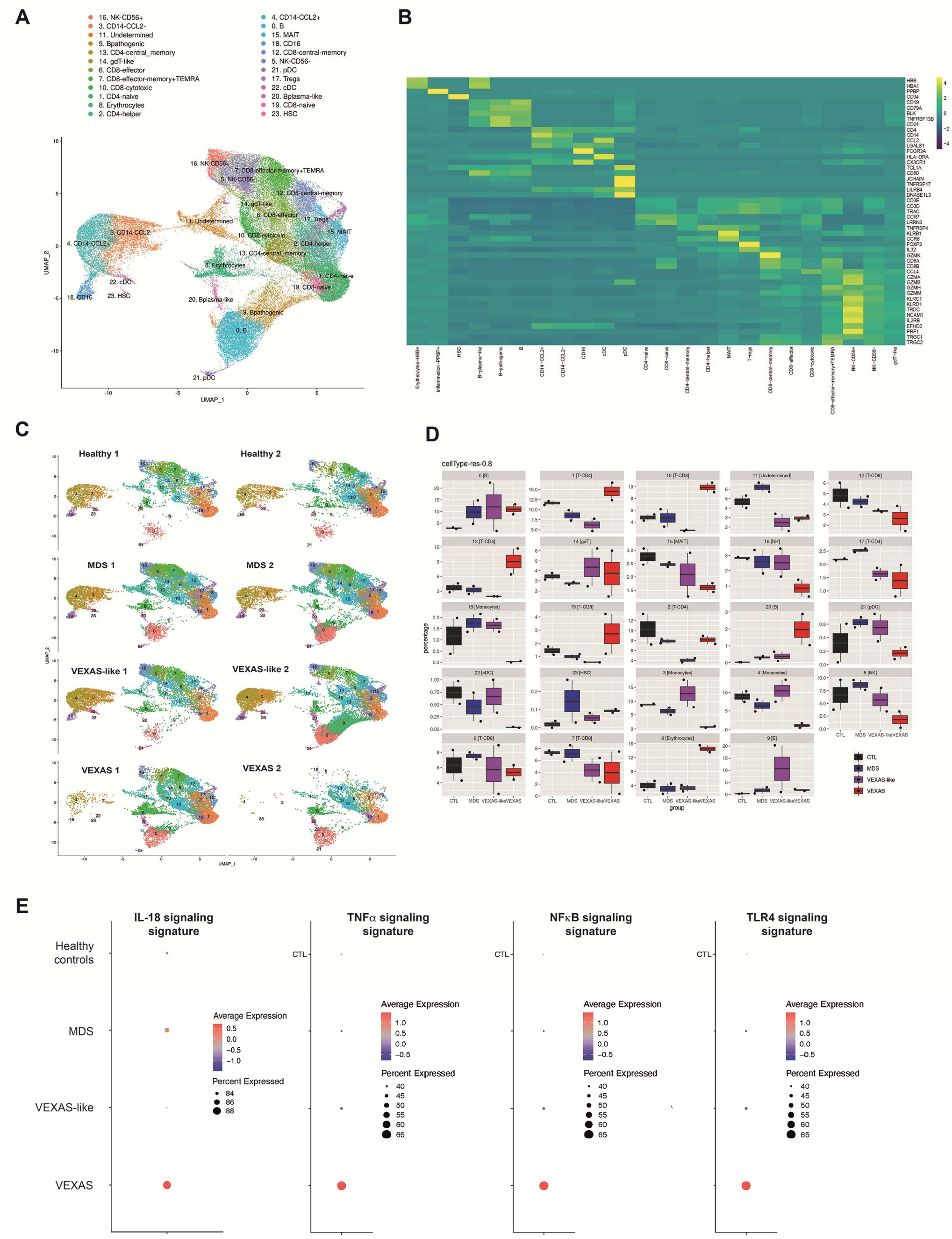

Supplementary Fig. 9. Visual representations of single-cell transcriptomic data of PBMCs in VEXAS syndrome.

(**A**) UMAP plots showing the projection of cell populations identified from PBMCs. (**B**) Heatmap of the normalized top differently expression genes in the different cell populations in PBMCs. (**C**) UMAP plots showing the projection of all PBMCs from patients with VEXAS, VEXAS-like, MDS and healthy controls. (**D**) Proportion (frequencies) of all different cell populations identified in the PBMCs of patients. (**E**) IL-18, TNF-α, NFκB and TLR4 signaling gene expression signatures in PBMCs from each patients' group. The size of the dot represents the percentage of cells in the clusters expressing the gene expression signature and the color intensity represents the average expression of the signature in that cluster.

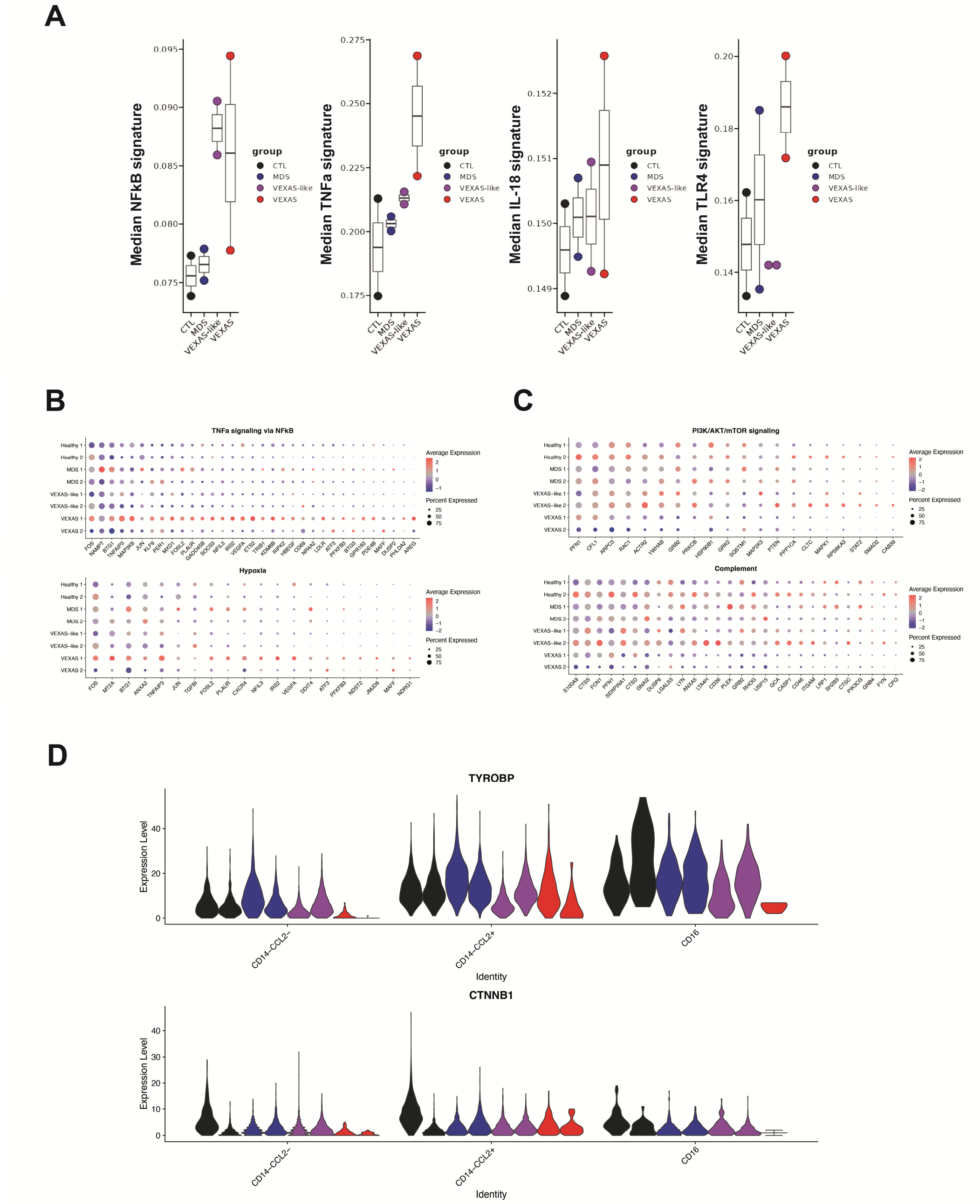

Supplementary Fig. 10. Individual representation of single-cell transcriptomic data of monocytes in VEXAS syndrome.

(**A**) IL-18, TNF-α, NFκB and TLR4 signaling gene expression signatures in monocytes from each patient. The size of the dot represents the percentage of cells in the clusters expressing the gene expression signature and the color intensity represents the average expression of the signature in that cluster. (**B**) Detailed analysis of the two most up-regulated pathways in monocytes from VEXAS, i.e. TNF-α signaling via NFκB pathway and hypoxia, from each patient. (**C**) Detailed analysis of the two most down-regulated pathways in monocytes from VEXAS, i.e. PI3K/AKT/mTOR signaling and complement pathways, from each patient. (**D**) Expression levels in each monocyte subsets of *TYROBP*, encoding for DAP12, and *CTNNB1*, encoding catenin beta-1,in from each patient.

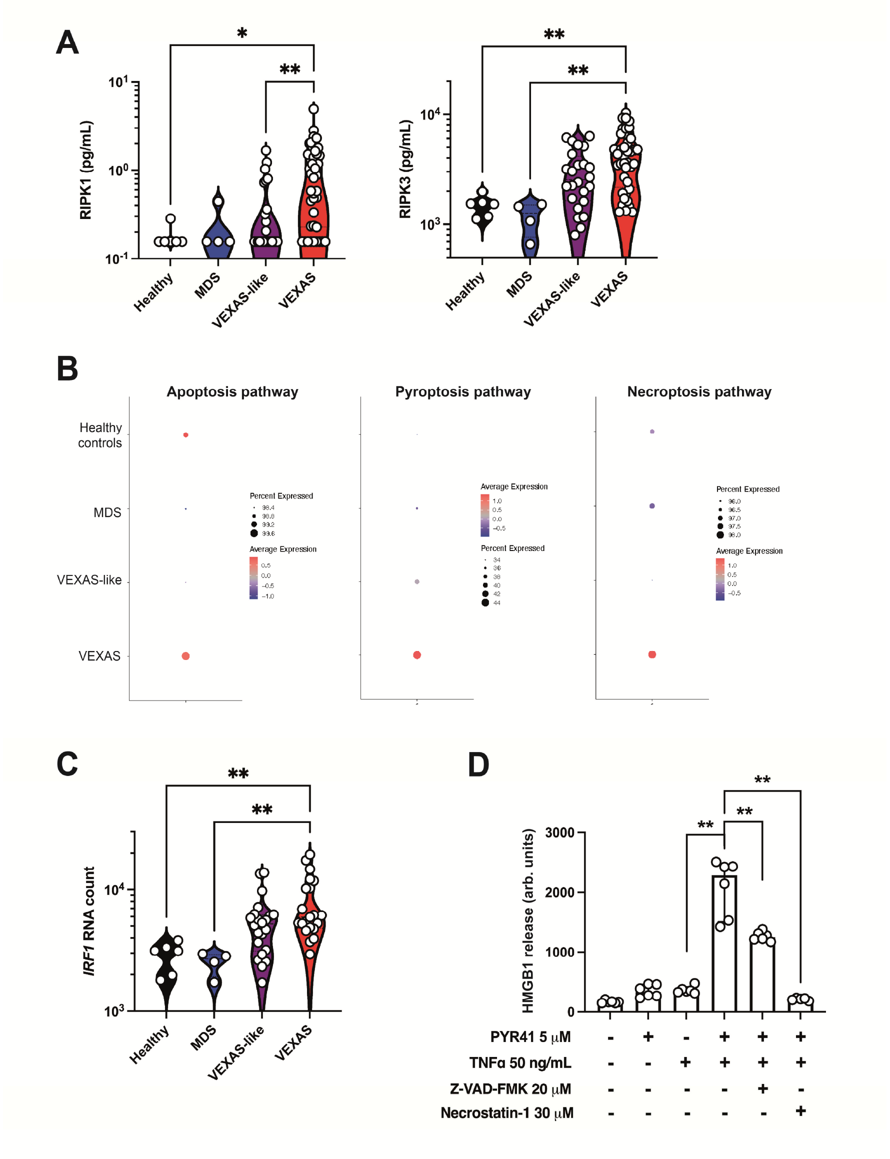

Supplementary Fig. 11. Inflammatory programmed cell death pathway in monocytes from VEXAS syndrome and inductions of caspase and RIPK1 dependent HMGB1 release in THP-1 cells.

(**A**) RIPK1 and RIPK3 plasma concentrations in patients with VEXAS, VEXAS-like, MDS and healthy controls. Each dot represents a single patient. (**B**) Apoptosis, pyroptosis and necroptosis gene expression signatures in monocytes from each patients' group. The size of the dot represents the percentage of cells in the clusters expressing the gene expression signature and the color intensity represents the average expression of the signature in that cluster. **(C)** Absolute RNA counts using Nanostring nCounter technology and showing decreased increased of *IRF1* transcripts in VEXAS. Each dot represents a single patient. (**D**) Co-treatment of PYR-41 and TNF- α Induces caspase and RIPK1 dependent HMGB1 release. THP-1-HMGB1-Lucia cells were pre-treated with a selective inhibitor of ubiquitin-activating enzyme E1 PYR-41 (5µM) with or without the pan-caspase inhibitor Z-VAD-FMK (20 µM) or RIPK1 inhibitor Necrostatin-1 (30µM) for 1 hour prior to incubation with recombinant human TNF-α (50 ng/ml). Z-VAD-FMK and Necrostatin were used respectively at 20µM and 30µM. Twenty-four hours later, the luciferase activity was determined by measuring the luminescence with QUANTI-Luc™ detection reagent (Invivogen) in a luminometer. **P ≤ 0.01. Analysis was performed using Mann-Whitney test. Data are shown as mean.

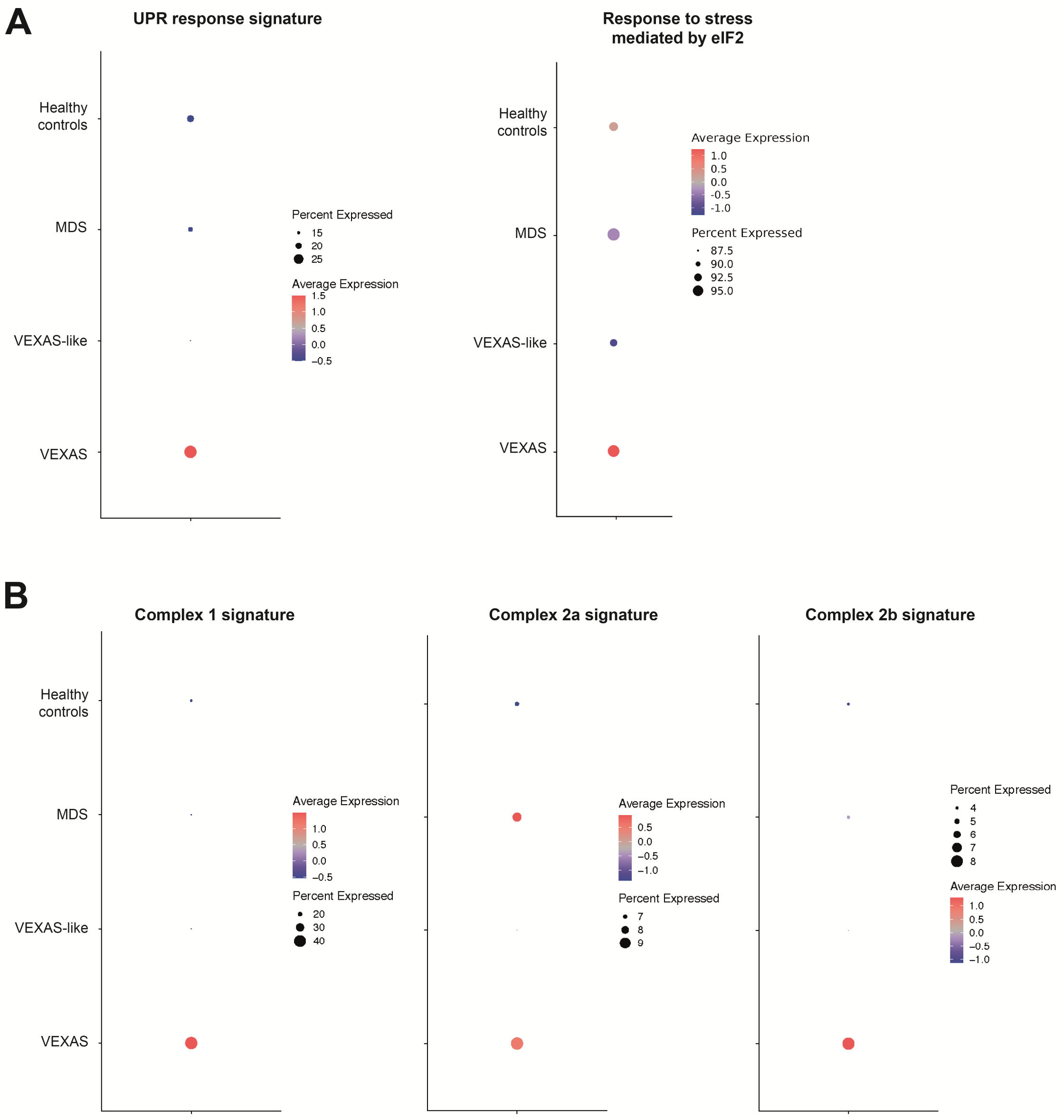

Supplementary Fig. 12. Unfolded protein response, response to stress mediated by EIF2 and complex 1, 2ab and 2b signatures in monocytes from VEXAS syndrome.
(**A**) **Unfolded protein response and response to stress mediated by EIF2** gene expression signatures in monocytes from each patients' group. (**B**) Signatures assessing the URP response and assembly of complexes involving RIPK1, i.e. complex 1, complex 2a and complex 2b, in monocytes from each patients' group. The size of the dot represents the percentage of cells in the clusters expressing the gene expression signature and the color intensity represents the average expression of the signature in that cluster.

Characteristics	Healthy controls	Myelodysplastic syndrome	VEXAS-like	VEXAS
**Participants – no.**	12	4	24	40
**Demographic characteristics**				
Male sex – no. (%)	12 (100)	4 (100)	24 (100)	40 (100)
Median age at onset (range) – yr	-	-	65 (43-82)	71 (50-89)
Median age at time analysis (range) – yr	60 (55-85)	76 (70-88)	70 (54-82)	74 (55-90)
**Genetic characteristics**				
Somatic *UBA1* variant (p.Met41) – no. (%)	0 (0)	0 (0)	0 (0)	40 (100)
p.Met41Thr (c.122T□C)	-	-	-	18 (45)
p.Met41Val (c.121A□G)	-	-	-	10 (25)
p.Met41Leu (c.121A□C)	-	-	-	6 (15)
Splice	-	-	-	6 (15)
**Key clinical features**				
Fever – no. (%)	0 (0)	0 (0)	9 (38)	27 (68)
Arthralgia/arthritis – no. (%)	0 (0)	0 (0)	16 (67)	26 (65)
Skin involvement – no. (%)	0 (0)	0 (0)	14 (58)	21 (53)
Pulmonary infiltrate – no. (%)	0 (0)	0 (0)	1 (4)	14 (35)
Ear and nose chrondritis – no. (%)	0 (0)	0 (0)	9 (38)	19 (48)
Venous thromboembolism – no. (%)	0 (0)	0 (0)	4 (17)	17 (43)
Ocular inflammation – no. (%)	0 (0)	0 (0)	3 (13)	12 (30)
Seritis – no. (%)	0 (0)	0 (0)	4 (17)	7 (18)
Macrocytic anemia – no. (%)	0 (0)	0 (0)	5 (21)	36 (90)
**Diagnostic or classification criteria that were met**				
Relapsing polychondritis – no. (%)	0 (0)	0 (0)	9 (38)	19 (48)
Sweet's syndrome – no. (%)	0 (0)	0 (0)	6 (25)	17 (43)
Myelodysplastic syndrome – no. (%)	0 (0)	4 (100)	10 (42)	21 (53)
Polyarteritis nodosa – no. (%)	0 (0)	0 (0)	1 (4)	4 (10)
Giant-cell arteritis/Polymyalgia rheumatica – no. (%)	0 (0)	0 (0)	5 (21)	3 (8)
**Laboratory findings at time analysis**				
Hemoglobin (IQR) – g/dL	15.7 (15.3-15.9)	11.6 (9.6-13.3)	11.6 (10.6-13.4)	10.0 (8.7-10.7)
Mean corpuscular volume (IQR) - fL	92 (89-94)	99 (92-105)	95 (91-99)	108 (100.5-114)
Leukocytes (IQR) – x 10^9/L	6.9 (6.2-8.3)	3.9 (2.4-5.7)	6.3 (4.1-7.8)	3.9 (2.5-5.6)
Neutrophils (IQR) – x 10^9/L	4.1 (3.1-5.0)	2.3 (1.5-2.9)	3.4 (2.0-6.4)	2.7 (1.4-3.8)
Lymphocytes (IQR) – x 10^9/L	2.4 (1.5-3.0)	1.1 (0.7-2.0)	1.2 (0.9-1.5)	0.8 (0.5-1.2)
Monocytes (IQR) – x 10^9/L	0.47 (0.30-0.76)	0.51 (0.17-0.73)	0.59 (0.42-0.80)	0.22 (0.14-0.31)
C-reactive protein (IQR) – mg/L	1.3 (0.0-3.5)	0.0 (0.0-0.8)	5.0 (1.7-19.5)	38.6 (13.8-83.0)
LDH (IQR) – UI/L	237 (213-252)	267 (224-286)	275 (218-338)	326 (255-426)
**Past treatment**				
Glucocorticoids	0 (0)	0 (0)	22 (92)	37 (93)
Synthetic DMARDs – no. (%)	0 (0)	0 (0)	15 (63)	22 (55)
Biologic or target synthetic DMARDS – no. (%)	0 (0)	0 (0)	13 (54)	23 (58)
5-azacytidine – no. (%)	0 (0)	0 (0)	2 (8)	9 (23)
**Current treatment at time analysis**				
Glucocorticoids – no. (%)	0 (0)	0 (0)	17 (71)	34 (85)
Median prednisone dose (IQR) – mg/day	0 (0)	0 (0)	10 (0-15)	10 (7-19)
Synthetic DMARDs – no. (%)	0 (0)	0 (0)	2 (8)	8 (20)
Biologic or target synthetic DMARDS – no. (%)	0 (0)	0 (0)	7 (29)	9 (23)
5-azacytidine – no. (%)	0 (0)	0 (0)	2 (8)	4 (10)
**Disease activity status at time analysis**				
Active disease – no. (%)	-	-	11 (46)	26 (65)
Inactive disease – no. (%)	-	-	13 (54)	14 (35)

DMARDs: disease-modifying antirheumatic drugs; IQR: interquartile range; LDH: lactate deshydrogenase ; MDS: myelodysplastic syndrome; VEXAS: vacuoles, E1 enzyme, X-linked, autoinflammatory, somatic; UBA1: Ubiquitin Like Modifier Activating Enzyme 1.
Laboratory values are indicated in median.

Supplementary Table 1. Demographic and clinical characteristics of participants included in the study.

**CD8+ T cells**	CD3+ CD66b- CD19- CD8+ CD4- CD14- CD161- TCR©™- CD123- CD11c-
**CD8 differentiation stages**	
Naive	CCR7+ CD27+ CD45RA+
Central memory	CCR7+ CD27+ CD45RA-
Effector memory	CCR7- CD27+
Terminal effector	CCR7- CD27-
**CD4+ T cells**	CD3+ CD66b- CD19- CD8- CD4+ CD14- CD161- TCR©™- CD123- CD11c
**CD4 differentiation stages**	
Naive	CCR7+ CD27+ CD45RA+
Central memory	CCR7+ CD27+ CD45RA-
Effector memory	CCR7- CD27+ CD45RA-
Terminal effector	CCR7- CD27- CD45RA-
**CD4 polarization stages**	
Th1	CXCR3+ CCR6- CCR4- CXCR5-
Th2	CXCR3- CCR6- CCR4+ CXCR5-
Th17	CXCR3- CCR6+ CCR4+ CXCR5-
Regulatory T cells	CD25+ CD127- CCR4+ HLA-DR-
**©™ T cells**	CD3+ CD66b- CD14- CD8dim CD4- TCR©™+
**Natural killer (NK) cells**	CD14- CD3- CD123- CD66b- CD45RA+ CD56+
**Mucosal-associated invariant T (MAIT) and NKT cells**	CD3+ CD66b- CD19- CD4- CD14- CD161+ TCR©™- CD28+ CD16-
**B cells**	CD3- CD14- CD56- CD16- CD19+ CD20+ HLA-DR+
**Plasmablasts**	CD3- CD14- CD16- CD66b- CD20- CD19+ CD56- CD38++ CD27++
**Dendritic cells (DC)**	
pDC	CD3- CD19- CD14- CD20- CD66b- HLA-DR+ CD11c- CD123+
mDC	CD3- CD19- CD14- CD20- HLA-DR+ CD11c+ CD123- CD16- CD38+ CD294-
**Monocytes**	CD3- CD19- CD56- CD66b- CD14+ HLA-DR+ CD11c+
Classical	CD14+ CD38+
Transitional	CD14low CD38 low
Non-classical	CD14- CD38-
**Neutrophils**	CD66b+ CD16+ HLA-DR-

Supplementary Table 2. Phenotypic definition of immune cell populations**.**

**Type II IFN (IFN-©)**	*CDKN1A, CXCL9, HLA-DMB, HLA-DPA1, HLA-DPB1, HLA-DRA, IDO1, JAK2, RARRES3, SLAMF7, SOCS1*
**TNF-〈**	*C3, CCL4, CD44, CD83, IRAK2, NFKB2, NFKBIA, RELB, SOCS3, SRC, TNFAIP3*
**IL-1®**	*CCL2, CCL20, CXCL2, IL1A, IL1B, IL6, LILRB1, NFKB1, NFKBIZ, POU2F2*
**IL-18**	*ARG1, B2M, BAX, BCL2, BID, CASP3, CASP8, CCL18, CCL19, CCL2, CCL20, CCL3, CCL4, CCL5, CD36, CD81, CD83, CEBPB, CHUK, CTNNB1, CXCL2, FADD, FAS, FN1, ICAM1, IFNG, IKBKB, IL10, IL12B, IL13, IL18, IL18R1, IL18RAP, IL1B, IL2RA, IL6, IL9, IRAK1, IRF1, ITGA2B, LCK, MAPK1, MYD88, NFKB1, NFKB2, NFKBIA, NFKBIZ, NOS2, PRKCD, PTGS2 RELA, SOCS3, SPP1, TBX21, TNF, TNFAIP3, TNFSF11, TP53, TRAF1, TRAF6*
**IL-6**	*CEBP, HRAS, IL6, IL6R, IL6ST, JAK1, JAK2, JAK3, RAF1, STAT3*
**NF⏐B**	*CHUK, FADD, IKBKB, IKBKG, IL1A, IL1R1, MYD88, NFKB1, NFKBIA, RELA, TNF, TNFAIP3, TNFRSF1B, TRAF6*

Supplementary Table 3. List of genes used to calculate zScores for each gene signature. Type I IFN, type II IFN, TNF-〈 and IL-1® signatures based on the study by Urrutia et al. (Cell Reports, 2016). IL-18 and IL-6 signatures based on Wikipathway. NF⏐B signature based on the Nanostring Immunology panel annotation file.

Top down-regulated gene in VEXAS vs healthy controls comparison (q-value <0.05)		
**Genes**	**estimate**	**q.value**
CCR2	-1,29091465	0,00059618
CSF1R	-1,26597199	3,3271E-06
CMKLR1	-1,16080021	0,00035086
CIITA	-1,10797175	0,00100311
MSR1	-1,07144846	0,00299568
CX3CR1	-1,03623855	5,718E-06
CASP10	-1,00440294	0,00358072
CD86	-0,94407751	0,00110443
CISH	-0,92904526	0,00110243
CXCR2	-0,90006158	0,00126545
**Top up-regulated gene in VEXAS vs healthy controls comparison (q-value <0.05)**		
**Genes**	**estimate**	**q.value**
PLAU	1,51719999	6,1809E-06
NFKBIA	1,18716352	1,0453E-08
LTF	1,17874396	0,00045728
TNFAIP3	1,08554059	3,105E-09
CTSG	1,07519794	0,00126741
CEACAM6	0,96033142	0,00188861
CXCL2	0,94350556	0,00125594
CEACAM8	0,91528168	0,00280908
CCL20	0,88154015	0,00088693
CD83	0,8808809	5,718E-06

Supplementary Table 4. Top down-regulated and up-regulated genes in VEXAS patients compared to healthy controls.

**Down-regulated genes common in VEXAS in comparison to healthy controls (q-value <0.05)**
IL16, CD244, TP53, IKBKB, NFATC3, MBP, RORC, IKBKAP, IL7R, CISH
**Up-regulated genes common in VEXAS in comparison to healthy controls (q-value <0.05)**
TNFAIP3, NFKBIA, RELB, NFKBIZ, CD83, PLAU, PSMD7, TRAF6, ICAM1, CTNNB1

Supplementary Table 5. Top down-regulated and up-regulated genes common in VEXAS and VEXAS like patients in comparison to healthy controls.

SampleID	pipeline	Status	nCells	nFeature_median	nCount_median	MT_median	Ribo_median
CONTROL1	SCT	Filtered	5466	1430	4482	6	29
CONTROL1	SCT	Pre-filtered	9426	949	2326	8	22
CONTROL2	SCT	Filtered	5814	1602	5352	5	35
CONTROL2	SCT	Pre-filtered	7887	1418	4584	6	29
MDS1	SCT	Filtered	10565	1185	3637	7	29
MDS1	SCT	Pre-filtered	19802	706	1691	10	22
MDS2	SCT	Filtered	12181	1162	3768	5	34
MDS2	SCT	Pre-filtered	16804	1002	3022	6	29
VEXAS1	SCT	Filtered	8986	1203	3918	6	36
VEXAS1	SCT	Pre-filtered	16802	653	1914	7	23
VEXAS2	SCT	Filtered	3668	924	2949	5	33
VEXAS2	SCT	Pre-filtered	8614	416	1556	6	12
VEXASLIKE1	SCT	Filtered	7624	1166	2799	4	19
VEXASLIKE1	SCT	Pre-filtered	10963	941	2057	4	16
VEXASLIKE2	SCT	Filtered	13689	1423	4198	7	31
VEXASLIKE2	SCT	Pre-filtered	15603	1353	3936	7	31

Supplementary Table 6 : Quality control parameters of scRNA-Seq data
