# Supplementary material for: VEXAS syndrome is characterized by inflammasome activation and monocyte dysregulation
Source: Nat Commun. 2024 Jan 30;15:910. doi: 10.1038/s41467-024-44811-4 (PMC10828464; doi:10.1038/s41467-024-44811-4)
Supplement: Supplementary file 2 — Description of Additional Supplementary Files [file 41467_2024_44811_MOESM2_ESM.pdf]

**Description of Additional Supplementary Files**

**Supplementary Data 1** : an excel file containing data on individual patients for biological and CyTOF data.

**Supplementary Data 2** : an excel file containing all the gene differentially expressed between VEXAS and Healthy patients comparison used in the figure 5.
