# Supplementary material for: VEXAS syndrome is characterized by inflammasome activation and monocyte dysregulation
Source: Nat Commun. 2024 Jan 30;15:910. doi: 10.1038/s41467-024-44811-4 (PMC10828464; doi:10.1038/s41467-024-44811-4)
Supplement: Supplementary file 5 — Reporting Summary [file 41467_2024_44811_MOESM5_ESM.pdf]

nature portfolio

Corresponding author(s): KOSMIDER
Last updated by author(s): December 20th, 2023

Reporting Summary

Nature Portfolio wishes to improve the reproducibility of the work that we publish. This form provides structure for consistency and transparency in reporting. For further information on Nature Portfolio policies, see our Editorial Policies and the Editorial Policy Checklist.

Please do not complete any field with "not applicable" or n/a. Refer to the help text for what text to use if an item is not relevant to your study.
For final submission: please carefully check your responses for accuracy; you will not be able to make changes later.

Statistics

For all statistical analyses, confirm that the following items are present in the figure legend, table legend, main text, or Methods section.

n/a	Confirmed	
☐	☑	The exact sample size (*n*) for each experimental group/condition, given as a discrete number and unit of measurement
☐	☑	A statement on whether measurements were taken from distinct samples or whether the same sample was measured repeatedly
☐	☑	The statistical test(s) used AND whether they are one- or two-sided<br>*Only common tests should be described solely by name; describe more complex techniques in the Methods section.*
☐	☑	A description of all covariates tested
☑	☐	A description of any assumptions or corrections, such as tests of normality and adjustment for multiple comparisons
☐	☑	A full description of the statistical parameters including central tendency (e.g. means) or other basic estimates (e.g. regression coefficient) AND variation (e.g. standard deviation) or associated estimates of uncertainty (e.g. confidence intervals)
☐	☑	For null hypothesis testing, the test statistic (e.g. *F*, *t*, *r*) with confidence intervals, effect sizes, degrees of freedom and *P* value noted<br>*Give P values as exact values whenever suitable.*
☑	☐	For Bayesian analysis, information on the choice of priors and Markov chain Monte Carlo settings
☑	☐	For hierarchical and complex designs, identification of the appropriate level for tests and full reporting of outcomes
☐	☑	Estimates of effect sizes (e.g. Cohen's *d*, Pearson's *r*), indicating how they were calculated

Our web collection on statistics for biologists contains articles on many of the points above.

Software and code

Policy information about availability of computer code

Data collection	GSE216548 contains all the files required to reproduce our analyse (FastQ and the processed data from Cell Ranger and the integration (rds object from Seura) https://www.ncbi.nlm.nih.gov/geo/query/acc.cgi?acc=GSE216548. All ,the other data are provided as source files with the manuscript.
Data analysis	For scRNA Seq, we indicated that based on raw UMI counts, we have selected the genes expressed in at least 3 cells and cells with at least 500 genes expressed. All the data collection/data analysis software/tools/algorithms/packages used in the study are clearly mentioned in the manuscript and are also listed here in the reporting summary (with version numbers).

For manuscripts utilizing custom algorithms or software that are central to the research but not yet described in published literature, software must be made available to editors and reviewers. We strongly encourage code deposition in a community repository (e.g. GitHub). See the Nature Portfolio guidelines for submitting code & software for further information.

Data

Policy information about availability of data

All manuscripts must include a data availability statement. This statement should provide the following information, where applicable:

- Accession codes, unique identifiers, or web links for publicly available datasets
- A description of any restrictions on data availability
- For clinical datasets or third party data, please ensure that the statement adheres to our policy

All the databases/datasets used in the study along with appropriately accessible links/accession codes in the manuscript under the "Data availability" section and in the source code file provided

Research involving human participants, their data, or biological material

Policy information about studies with human participants or human data. See also policy information about sex, gender (identity/presentation), and sexual orientation and race, ethnicity and racism.

Reporting on sex and gender	All male patients according to the initial description of VEXAS syndrom in the NEJM (Beck et al, 2020)
Reporting on race, ethnicity, or other socially relevant groupings	N/A
Population characteristics	detailled in supplementary table 1
Recruitment	All the refered patients to Cochin Hospital for UBA1 testing between January 2021 and May 2021 were included as mentionned in the MS. They all have signed the informed consent. There is no biais of selection in this exploratory study.
Ethics oversight	As mentionned in the MS (line 528), we have an ethical approvment (Number AA-2021-08040)

Note that full information on the approval of the study protocol must also be provided in the manuscript.

Field-specific reporting

Please select the one below that is the best fit for your research. If you are not sure, read the appropriate sections before making your selection.

[x] Life sciences [ ] Behavioural & social sciences [ ] Ecological, evolutionary & environmental sciences

For a reference copy of the document with all sections, see nature.com/documents/nr-reporting-summary-flat.pdf

Life sciences study design

All studies must disclose on these points even when the disclosure is negative.

Sample size	80 samples. This exploratory study was not based on a predefined sample size.
Data exclusions	Only patients positive for SARS-Cov2 infection were excluded as mentionned in the MS
Replication	No replication was made in this exploratory study. However, UBA1 mutationnal testing made by Sanger sequencing and confirmed ny NGS could be considered as a replication
Randomization	According to genotype (with or without UBA1 mutation for patients)
Blinding	All the experiments were performed without the kowledge of the presence of an UBA1 mutation. The analyses were made according to the UBA1 mutationnal status to obtain separated groups.

Behavioural & social sciences study design

All studies must disclose on these points even when the disclosure is negative.

Study description	N/A
Research sample	N/A
Sampling strategy	N/A
Data collection	N/A
Timing	N/A
Data exclusions	N/A
Non-participation	N/A
Randomization	N/A

Ecological, evolutionary & environmental sciences study design

All studies must disclose on these points even when the disclosure is negative.

Study description	N/A
Research sample	N/A
Sampling strategy	N/A
Data collection	N/A
Timing and spatial scale	N/A
Data exclusions	N/A
Reproducibility	N/A
Randomization	N/A
Blinding	N/A

Did the study involve field work? ☐ Yes ☒ No

Field work, collection and transport

Field conditions	N/A
Location	N/A
Access & import/export	N/A
Disturbance	N/A

Reporting for specific materials, systems and methods

We require information from authors about some types of materials, experimental systems and methods used in many studies. Here, indicate whether each material, system or method listed is relevant to your study. If you are not sure if a list item applies to your research, read the appropriate section before selecting a response.

Materials & experimental systems

n/a	Involved in the study	
☐	☒	Antibodies
☐	☒	Eukaryotic cell lines
☒	☐	Palaeontology and archaeology
☒	☐	Animals and other organisms
☐	☒	Clinical data
☒	☐	Dual use research of concern
☒	☐	Plants

Methods

n/a	Involved in the study	
☒	☐	ChIP-seq
☐	☒	Flow cytometry
☒	☐	MRI-based neuroimaging

Antibodies

Antibodies used	All the CyTOF antibodies are detailled in the MS and the gating strategies are indicated in suppl material
Validation	As indicated in the MS, the panel of CyTOF antiboidies is a commercial panel already validated and used by the team in Hadjadj et al, Science 2020, PMID 32661059

Eukaryotic cell lines

Policy information about cell lines and Sex and Gender in Research

Cell line source(s)	THP1 (Tohoku Hospital Pediatrics-1) Cells-HMGB1-Lucia™, karyotype XY, InvivoGen, San Diego, CA, USA, catalog reference thp-gb1lc.
Authentication	Certificates of Origin and authentication is guaranteed by the supplier. Informations on https://www.invivogen.com/thp1-hmgb1-lucia
Mycoplasma contamination	THP1-HMGB1-Lucia™ cells are guaranteed mycoplasma-free by the supplier and has not been subsequently tested.
Commonly misidentified lines (See ICLAC register)	N/A

Palaeontology and Archaeology

Specimen provenance	
Specimen deposition	
Dating methods	

☐ Tick this box to confirm that the raw and calibrated dates are available in the paper or in Supplementary Information.

Ethics oversight	

Note that full information on the approval of the study protocol must also be provided in the manuscript.

Animals and other research organisms

Policy information about studies involving animals; ARRIVE guidelines recommended for reporting animal research, and Sex and Gender in Research

Laboratory animals	
Wild animals	
Reporting on sex	
Field-collected samples	
Ethics oversight	

Note that full information on the approval of the study protocol must also be provided in the manuscript.

Clinical data

Policy information about clinical studies

All manuscripts should comply with the ICMJE guidelines for publication of clinical research and a completed CONSORT checklist must be included with all submissions.

Clinical trial registration	This exploraty study was supported by CARPEM/RADIPEM authorization<br>as mentionned here https://carpem.fr/activites/les-plateformes/biobanques/projets-de-recherche-collection-oncocentre/
Study protocol	Exploratory study without clinical intervention
Data collection	As inidcated here https://carpem.fr/activites/les-plateformes/biobanques/projets-de-recherche-collection-oncocentre/
Outcomes	N/A

Dual use research of concern

Policy information about dual use research of concern

Hazards

Could the accidental, deliberate or reckless misuse of agents or technologies generated in the work, or the application of information presented in the manuscript, pose a threat to:

No	Yes	
[ ]	[X]	Public health
[X]	[ ]	National security
[X]	[ ]	Crops and/or livestock
[X]	[ ]	Ecosystems
[X]	[ ]	Any other significant area

Experiments of concern

Does the work involve any of these experiments of concern:

No	Yes	
[X]	[ ]	Demonstrate how to render a vaccine ineffective
[X]	[ ]	Confer resistance to therapeutically useful antibiotics or antiviral agents
[X]	[ ]	Enhance the virulence of a pathogen or render a nonpathogen virulent
[X]	[ ]	Increase transmissibility of a pathogen
[X]	[ ]	Alter the host range of a pathogen
[X]	[ ]	Enable evasion of diagnostic/detection modalities
[X]	[ ]	Enable the weaponization of a biological agent or toxin
[X]	[ ]	Any other potentially harmful combination of experiments and agents

Plants

Seed stocks

Novel plant genotypes

Authentication

ChIP-seq

Data deposition

[ ] Confirm that both raw and final processed data have been deposited in a public database such as GEO.

[ ] Confirm that you have deposited or provided access to graph files (e.g. BED files) for the called peaks.

Data access links
May remain private before publication.

Files in database submission

Genome browser session
(e.g. UCSC)

Methodology

Replicates

Sequencing depth

Antibodies

Peak calling parameters

Data quality

Software

Flow Cytometry

Plots

Confirm that:

- [X] The axis labels state the marker and fluorochrome used (e.g. CD4-FITC).
- [X] The axis scales are clearly visible. Include numbers along axes only for bottom left plot of group (a 'group' is an analysis of identical markers).
- [X] All plots are contour plots with outliers or pseudocolor plots.
- [X] A numerical value for number of cells or percentage (with statistics) is provided.

Methodology

Sample preparation	As described by the provider of the Human Immunology_v2 kit
Instrument	As indicated in the MS
Software	FlowJo as indicated in the MS
Cell population abundance	According to each sample.
Gating strategy	Shown in the MS (Suppl Fig 2)

- [X] Tick this box to confirm that a figure exemplifying the gating strategy is provided in the Supplementary Information.

Magnetic resonance imaging

Experimental design

Design type	
Design specifications	
Behavioral performance measures	
Imaging type(s)	
Field strength	
Sequence & imaging parameters	
Area of acquisition	

Diffusion MRI [ ] Used [ ] Not used

Preprocessing

Preprocessing software	
Normalization	
Normalization template	
Noise and artifact removal	
Volume censoring	

Statistical modeling & inference

Model type and settings	
Effect(s) tested	

Specify type of analysis: [ ] Whole brain [ ] ROI-based [ ] Both

Statistic type for inference

(See Eklund et al. 2016)

Correction

Models & analysis

n/a	Involved in the study
X	☐ Functional and/or effective connectivity
X	☐ Graph analysis
X	☐ Multivariate modeling or predictive analysis

Functional and/or effective connectivity

Graph analysis

Multivariate modeling and predictive analysis

This checklist template is licensed under a Creative Commons Attribution 4.0 International License, which permits use, sharing, adaptation, distribution and reproduction in any medium or format, as long as you give appropriate credit to the original author(s) and the source, provide a link to the Creative Commons license, and indicate if changes were made. The images or other third party material in this article are included in the article's Creative Commons license, unless indicated otherwise in a credit line to the material. If material is not included in the article's Creative Commons license and your intended use is not permitted by statutory regulation or exceeds the permitted use, you will need to obtain permission directly from the copyright holder. To view a copy of this license, visit http://creativecommons.org/licenses/by/4.0/
