# Supplementary material for: VEXAS syndrome is characterized by inflammasome activation and monocyte dysregulation
Source: Nat Commun. 2024 Jan 30;15:910. doi: 10.1038/s41467-024-44811-4 (PMC10828464; doi:10.1038/s41467-024-44811-4)
Supplement: Supplementary file 6 — Source Data [file 41467_2024_44811_MOESM6_ESM.zip › Source_DATA_KOSMIDER/Description_source_data_KOSMIDER.docx]

This ZIP Source Data files contains

Supplementary Data 1 : an excel file containing data on individual patients for biological and CyTOF data. This file is notified in the text.

Supplementary Data 2 : an excel file containing all the gene differentially expressed between VEXAS and Healthy patients comparison used in the figure 5. This file is notified in the text.

The ZIP file Source Data files contains also 6 folders containint all the raw individual data used to generate the figures and the supplementary.

The Folders Raw_DATA_KOSMIDER_Fig1, 2 4 and 5 contain all the individual raw data obtained and used to generate main and supplemental figures

The Folder Raw_DATA_KOSMIDER_Fig3 contain all the raw data obtained from the comparison ofdifferential expression between VEXAS Lesional vs Normal in RNA-Seq processing

The Folder Raw_DATA_KOSMIDER_Fig6 contain all the comparisons made according to the scRNA-Seq whom raw files are already available (GSE216548)
